# Differential modulation of polycystin-2 gain-of-function channels by cysteine-reactive compounds, amphiphilic substances, and S4-S5 linker mutations

**DOI:** 10.1016/j.jbc.2025.110766

**Published:** 2025-09-24

**Authors:** Linda Geiges, Tobias Staudner, Juthamas Khamseekaew, Christoph Korbmacher, Alexandr V. Ilyaskin

**Affiliations:** Institute of Cellular and Molecular Physiology, Friedrich-Alexander-Universität Erlangen-Nürnberg, Erlangen, Germany

**Keywords:** autosomal-dominant polycystic kidney disease (ADPKD), *renal physiology*, polycystin-2, *transient receptor potential channels (TRP channels)*, *electrophysiology*, S4-S5 linker, *site-directed mutagenesis*, *Xenopus*, *oocyte*, amphiphilic modulator

## Abstract

Polycystin-2 (PC2) mutations are responsible for ∼15% of cases of autosomal-dominant polycystic kidney disease (ADPKD). PC2 belongs to the transient receptor potential ion channel family and can function as a homotetrameric nonselective cation channel. Little is known about its gating mechanism, and no specific PC2 activators or inhibitors have been identified. In this study, we performed a comparative electrophysiological analysis of two well-established gain-of-function PC2 constructs, PC2 F604P and PC2 L677A N681A, expressed in *Xenopus laevis* oocytes. Interestingly, PC2 F604P, but not PC2 L677A N681A, could be inhibited by several membrane-permeable cysteine-reactive compounds. In contrast, positively charged cysteine-reactive compounds had no inhibitory effect. Thus, the inhibitory effect probably involves covalent modification of intracellular cysteine residues. Consistent with this, site-directed mutagenesis revealed a critical functional role of the free cysteine residue C593, localized in the intracellular S4-S5 linker of PC2 F604P. Moreover, the ion channel function of PC2 F604P was disturbed by S4-S5 linker mutations flanking C593. Interestingly, several structurally unrelated amphiphilic substances mimicked the inhibitory effect of membrane-permeable cysteine-reactive compounds on PC2 F604P without affecting PC2 L677A N681A. Collectively, our data suggest a critical role of the S4-S5 linker and the plasma membrane lipid environment in F604P-triggered PC2 gating. Finally, we demonstrated that the ADPKD-associated mutation located within the S4-S5 linker (N580K) completely abolished or significantly reduced currents mediated by PC2 F604P or PC2 L677A N681A, respectively. These findings provide new insights into molecular mechanisms involved in PC2 gating.

Autosomal-dominant polycystic kidney disease (ADPKD) is the most common monogenic renal disease, with a prevalence of approximately one in 1000 individuals (1). The disease is characterized by progressive formation and growth of renal cysts, which ultimately leads to end-stage renal disease (ESRD). In most cases, ADPKD is caused by mutations in the genes encoding either polycystin-1 (PC1) or polycystin-2 (PC2), accounting for ∼80% or ∼15% of cases, respectively. While the disease is inherited in an autosomal-dominant manner, it is recessive at the cellular level and requires a somatic “second-hit” mutation to initiate cyst development (2, 3, 4, 5, 6, 7). Although PC1 and PC2 were identified almost 30 years ago (8, 9), their physiological function and role in ADPKD pathogenesis remain incompletely understood.

PC2 belongs to the transient receptor potential (TRP) family of nonselective cation channels (10). As one of the founding members of the TRPP (polycystin) subfamily, PC2 is also known as TRPP1 (formerly TRPP2). Controversial findings regarding PC2 function as an ion channel have been reported. The commonly accepted paradigm implies that the pathogenically relevant function of PC2 is linked to the primary cilium, where it colocalizes with PC1 (11, 12, 13). However, PC2 was also found in the endoplasmic reticulum (ER), where it was suggested to facilitate Ca^2+^ release either directly acting as a calcium channel or indirectly *via* interaction with IP_3_ or RyR receptors (14, 15, 16, 17, 18). Intriguingly, a recent study suggests that PC2 may function as a potassium channel in the ER to facilitate potassium-calcium counterion exchange (19). In this study, *Padhy et al.* showed that expression of another potassium channel, TRIC-B, could functionally replace PC2 in the ER and ameliorate the cystic phenotype in a mouse model with PC2 deficiency (19).

PC2 has a typical TRP ion channel structure with six transmembrane domains (S1–S6) and cytosolic C- and N-termini. Recently published cryo-EM structures of PC2 demonstrated that four PC2 subunits can form a homotetrameric ion channel with a domain swapping architecture (20, 21, 22, 23, 24, 25). Furthermore, recent structural and functional evidence suggested that PC2 can also form heterotetrameric ion channels in complex with PC1, exhibiting a 3:1 stoichiometry (26, 27, 28, 29, 30). According to the available structural information, each PC2 subunit is composed of a voltage sensor–like domain (VSLD) formed by the first four transmembrane domains (S1–S4). The VSLD of PC2 resembles that of other TRP channels and is structurally related to the voltage sensor domain of voltage-gated ion channels (31). The fifth (S5) and the sixth (S6) transmembrane domain form the pore domain (PD) of PC2. On the intracellular side, a short α-helical S4-S5 linker runs parallel to the lipid bilayer and connects the VSLD and PD. Another feature of PC2 is a large extracellular, highly structured domain called “polycystin domain” (20) or “tetragonal opening for polycystins” (TOP) (21, 22) domain, which is localized between the S1 and S2 transmembrane domains and forms extensive interactions with the adjacent subunit (32).

The absence of specific natural or pharmacological activators and inhibitors of PC2 substantially complicates its functional characterization, particularly in native tissues but also in expression systems. Indeed, when WT PC2 is heterologously expressed, it is difficult to detect reliably PC2-mediated whole-cell currents probably because the ion channel remains largely silent at the plasma membrane with a very low open probability (27, 33, 34). So far, little is known about the gating mechanism of PC2 and about potential stimuli that may activate the channel. Interestingly, PC2 opening can be triggered artificially by introducing a proline substitution into the S5 transmembrane domain (F604P; (33)). Structural data of this artificial gain-of-function (GOF) PC2 mutant indicated that the F604P mutation leads to a lateral shift of the S4-S5 linker and to a π-to-α helix transition in the S6 domain resulting in a combined twisting and bending motion of this domain (23). This leads to the opening of the channel's lower gate by removing the pore blocking residues L677 and N681 from the ion translocation pathway (23). In contrast, the opposite transition, an α-to-π helix transition, has been reported for several TRPV channels as their natural activating mechanism (35, 36, 37, 38, 39, 40, 41). It has been proposed that the π-to-α conformational change produced by the F604P mutation may mimic the natural gating mechanism of PC2 (42). This justified research efforts to study functional effects of ADPKD-associated mutations using this PC2 construct (33, 34, 43). Alternatively, PC2 currents can be elicited by replacing the pore blocking residues L677 and N681 with alanines (L677A N681A; (27)). This likely results in a constitutive activation of the channel without substantial conformational changes in the transmembrane domains. Thus, these two GOF PC2 constructs differ fundamentally in their mode of action. Both GOF constructs were used in our recent study to reveal distinct functional effects of three ADPKD-associated pore mutations and to investigate the underlying molecular mechanisms (34).

In the present study, we performed a comparative electrophysiological analysis of PC2 F604P *versus* PC2 L677A N681A to gain new insights into the molecular mechanisms involved in PC2 gating. Collectively, our observations indicate an important role of the S4-S5 linker and lipid–protein interactions in F604P-induced channel opening. Furthermore, we demonstrated that the ADPKD-associated N580K mutation localized within the S4-S5 linker exerted a complete loss-of-function (LOF) effect on PC2 F604P but did not fully inhibit the ion channel function of PC2 L677A N681A. Our study provides novel insights into the mechanisms of PC2 ion channel function at the molecular level and has potential (patho-)physiological implications.

## Results

### Membrane-permeable cysteine-reactive methanethiosulfonate reagents and NEM have an inhibitory effect on PC2 F604P but not on PC2 L677A N681A ion channel function

**Figure 1:**
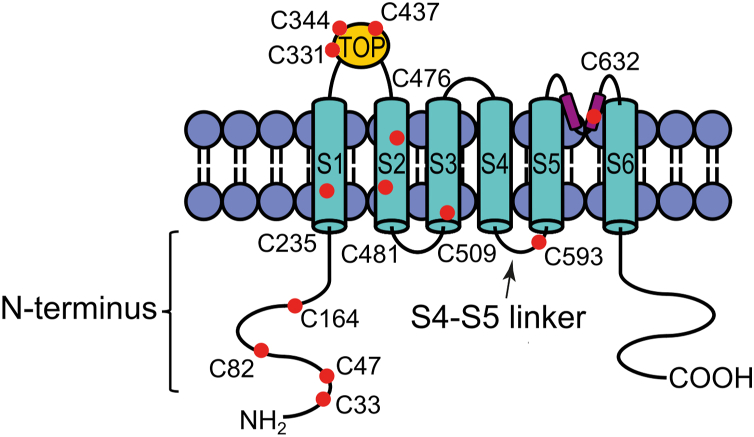
**Topology of PC2 subunit in the lipid bilayer.** Localization of cysteine residues in different PC2 domains is highlighted with *red dots*. S1 to S6: transmembrane domains 1 to 6. PC2, polycystin-2.

**Figure 2:**
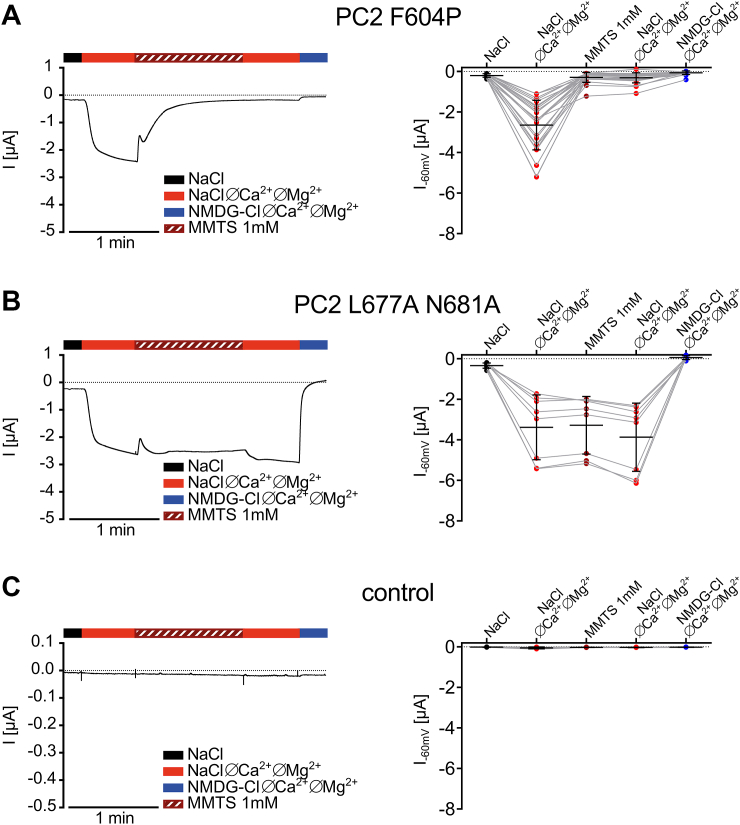
**MMTS acutely inhibits PC2 F604P but not PC2 L677A N681A.***Left panels:* representative whole-cell current traces obtained from an oocyte expressing PC2 F604P (*A*) or PC2 L677A N681A (*B*) or from a control oocyte (*C*) clamped at −60 mV. Different bath solutions were applied as indicated by *bars*. MMTS (1 mM) was applied in a NaCl ØCa^2+^ØMg^2+^ bath solution. Note that the brief transient initial inhibition of PC2 mediated currents in response to MMTS exposure is probably an artifact caused by the solution exchange and minor contamination of the superfusing solution with divalent cations. *Right panels:* summary data from similar experiments as shown in the corresponding *left panels*. Inward current values reached in each extracellular solution as indicated are shown. Mean ± SD and individual data points are shown (a: N = 3, n = 26; b: N = 1, n = 8; c: N = 2, n = 6; N indicates the number of different batches of *Xenopus laevis* oocytes, and n indicates the number of individual oocytes analyzed per experimental group). Lines connect data points obtained from the same oocyte. *Dotted line* indicates zero current level. PC2, polycystin-2; MMTS, methyl methanethiosulfonate.

We hypothesized that free cysteine residues scattered over different PC2 domains can be targeted and modified by thiol-reactive reagents. With this approach we aimed to identify functionally important PC2 domains and uncover functional differences between two PC2 GOF mutants. PC2 contains eleven free cysteine residues localized in different domains of the channel (Fig. 1). In addition, two cysteine residues (C331, C344) form a disulfide bridge in the TOP domain of the channel (32). In case chemical modification of a cysteine residue occurs in a functionally important channel region, it may alter PC2 ion channel function. Indeed, a small membrane-permeant thiol-reactive substance methyl methanethiosulfonate (MTS) (methyl-MTS, MMTS) has previously been used as a tool to identify functionally important cysteine residues in ion channels and membrane transporters (44, 45, 46, 47). Therefore, we investigated whether MMTS can modulate the ion channel function of PC2 F604P or PC2 L677A N681A, heterologously expressed in *Xenopus laevis* oocytes. PC2-mediated currents were assessed using the two-electrode voltage clamp technique (TEVC) essentially as described previously (34, 48). To stimulate PC2-mediated sodium inward currents at a negative holding potential, divalent cations (Ca^2+^, Mg^2+^) were removed from the bath solution. As illustrated by representative current traces and summary data from oocytes expressing PC2 F604P (Fig. 2*A*) or PC2 L677A N681A (Fig. 2*B*), divalent cation removal elicited a substantial inward current in these oocytes which was absent in control oocytes (Fig. 2*C*). This was consistent with previous findings (23, 27, 33, 34, 48) and confirmed functional expression of the two GOF PC2 mutants. Importantly, in the PC2 F604P expressing oocytes application of 1 mM MMTS largely inhibited this inward current in divalent cation-free bath solution (Fig. 2*A*). Indeed, after approximately 1 min of MMTS treatment, the PC2 F604P mediated inward currents were nearly abolished. Subsequent washout of MMTS did not result in a recovery of the currents, indicating that the inhibitory effect of MMTS is irreversible. Substituting Na^+^ with the large organic cation NMDG^+^ at the end of the recording caused only a marginal additional reduction of the inward currents, consistent with our interpretation that MMTS inhibited the channel almost completely. Surprisingly, the currents measured in PC2 L677A N681A expressing oocytes were largely unaffected by MMTS application (Fig. 2*B*). In contrast, switching from sodium-containing to NMDG^+^-containing extracellular solution largely abolished the PC2 L677A N681A mediated inward currents as expected, confirming that they were predominantly carried by Na^+^. In control oocytes baseline inward currents were very small and essentially unaffected by corresponding bath solution changes (Fig. 2*C*).

**Figure 3:**
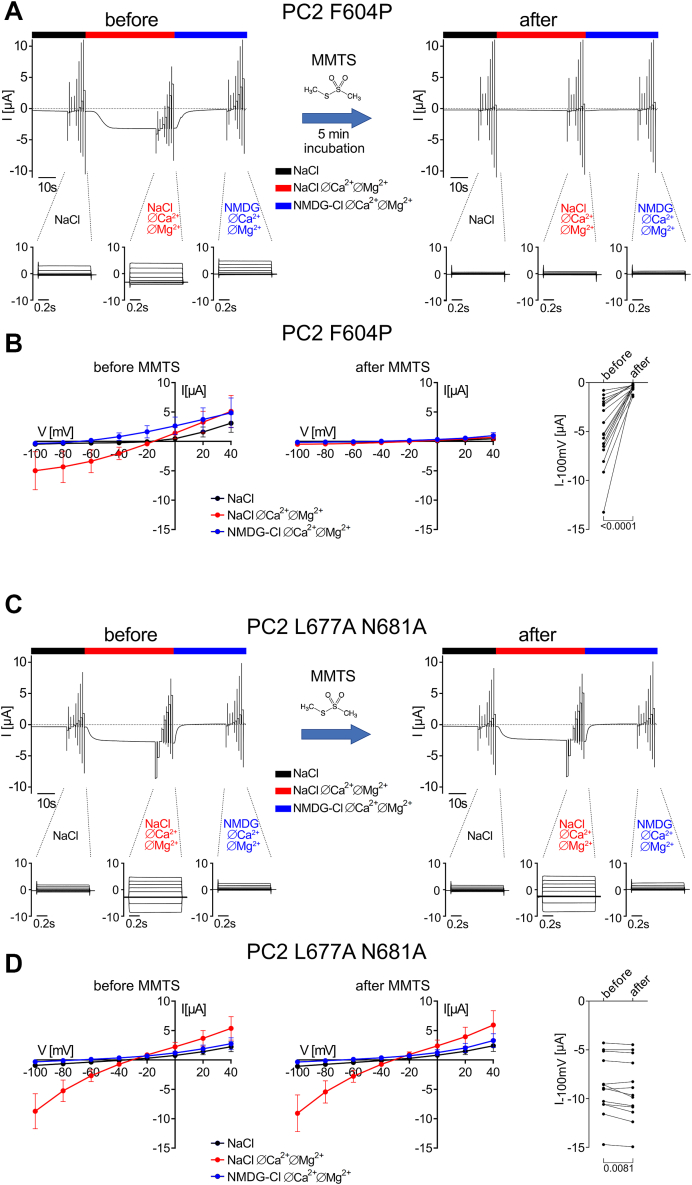
**PC2 F604P but not PC2 L677A N681A was strongly and irreversibly inhibited by incubation with MMTS (1 mM).***A* and *C,* representative whole-cell current traces obtained in an oocyte expressing PC2 F604P (*A*) or PC2 L677A N681A (*C*) before (*left panels*) and after (*right panels*) 5 min incubation in ND9 solution supplemented with MMTS (1 mM). Application of different bath solutions during the current measurement is indicated by *bars*. For each condition, a voltage step protocol was performed with consecutive 1000 ms voltage steps in 20 mV increments starting with a hyperpolarizing pulse to −100 mV from a holding potential of −60 mV. Overlays of the corresponding whole-cell current traces are shown below the traces for each bath solution. The oocyte was not clamped during the incubation with MMTS. Before the second current measurement, MMTS was washed out with NaCl bath solution. Impaling microelectrodes were not removed from the oocyte until the end of the experiment. *B* and *D, left* and *middle panels:* average I/V plots (mean ± SD) were constructed from similar experiments as shown in (*A*) and (*C*) using the mean current values measured during the last 300 ms of the voltage pulses. Data points represent average values from 13 to 18 oocytes (*B*: N = 5, n = 18; *D*: N = 4, n = 13; N indicates the number of different batches of *Xenopus laevis* oocytes, and n indicates the number of individual oocytes analyzed per experimental group). *Right panels:* summary data from the same experiments shown in *left* and *middle panels* demonstrate maximal inward current values reached during application of hyperpolarizing pulses of −100 mV in NaCl ØCa^2+^ØMg^2+^ solution before and after MMTS application. Lines connect data points obtained from the same oocyte. *p* values are calculated using Wilcoxon matched-pairs signed rank test. PC2, polycystin-2; MMTS, methyl methanethiosulfonate.

To estimate the effect of MMTS at different holding potentials and to reduce the amount of reagent needed, we modified the experimental protocol for all subsequent experiments. As shown in Figure 3, PC2-mediated currents were determined in each individual oocyte twice: before and after a 5-min incubation with MMTS. Consistent with the results obtained in experiments with continuous application of MMTS (Fig. 2*A*), PC2 F604P-mediated inward and outward currents were almost completely (by about 90%) and irreversibly abolished after MMTS incubation (Fig. 3, *A* and *B*). In contrast, PC2 L677A N681A-mediated currents remained largely unaffected (Fig. 3, *C* and *D*). Similarly, no effect of MMTS was observed in control oocytes (Fig. S1).

**Figure 4:**
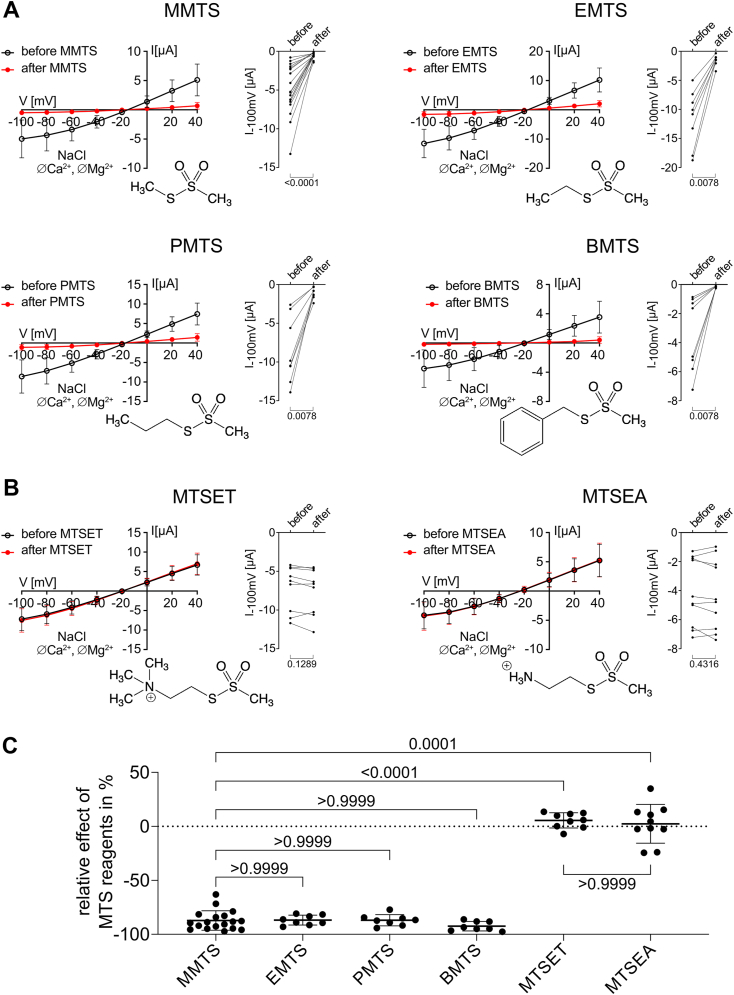
**Lipophilic but not positively charged MTS reagents inhibit PC2 F604P.***A* and *B,* whole-cell currents in oocytes expressing PC2 F604P were detected before and after 5 min incubation with different lipophilic (*A*) or positively charged (*B*) MTS substances (1 mM). Experiments were performed as described in Figure 3. The MMTS data are the same as shown in Figure 3, *A* and *B*. *Left panels:* average I/V plots (mean ± SD) obtained in NaCl ØCa^2+^ØMg^2+^ solution before and after incubation with the MTS reagent as indicated. Chemical structures of the corresponding substances are shown below the corresponding I/V plots. *Right panels:* summary data from the experiments shown in the corresponding *left panels* depict maximal inward current values reached at −100 mV before and after incubation with the respective MTS reagent. Lines connect data points obtained from the same oocyte. *p* values were calculated using Wilcoxon matched-pairs signed rank test. *C,* relative effect of MTS reagents in % calculated using the current values shown in *right panels* in (*A*) and (*B*): (I−100mV[after]I−100mV[before]−1)×100%. *p* values were calculated using Kruskal–Wallis and Dunn's multiple comparisons test (N = 5, n = 18 for MMTS as in Figure 3*B*; N = 2, n = 8–10 for all other MTS substances; N indicates the number of different batches of *Xenopus laevis* oocytes, and n indicates the number of individual oocytes analyzed per experimental group). PC2, polycystin-2; MMTS, methyl methanethiosulfonate; MTS, methanethiosulfonate.

Next, we assessed whether other cysteine-reactive MTS reagents, lipophilic ethyl methanethiosulfonate, propyl methanethiosulfonate, and benzyl methanethiosulfonate (BMTS) or hydrophilic, positively charged 2-(trimethylammonium)ethyl methanethiosulfonate (MTSET) and 2-aminoethyl methanethiosulfonate (MTSEA), had similar effects on PC2 currents as MMTS. Importantly, all tested lipophilic reagents strongly inhibited PC2 F604P currents to a similar extent as MMTS (Fig. 4, *A* and *C*), whereas charged MTS reagents produced no significant inhibitory effect (Fig. 4, *B* and *C*). In additional experiments, we demonstrated that an intact thiol-reactive group in MMTS is required for its inhibitory effect. Indeed, the MMTS-mediated inhibition of PC2 F604P was prevented when the MMTS-containing solution was supplemented with the reducing agent DTT (30 mM) (Fig. S2). DTT likely quenched MMTS before any modification of PC2 could occur. However, after inhibiting PC2 F604P with MMTS, this inhibition could not be reversed by subsequent application of DTT (Fig. S3).

**Figure 5:**
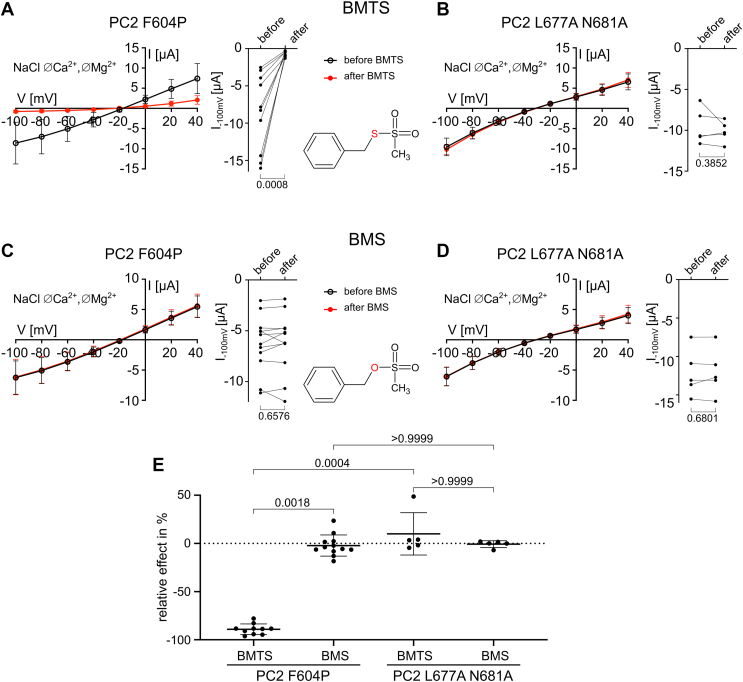
**The inhibitory effect of BMTS on PC2 F604P was abolished when its cysteine-reactive MTS group was replaced by an MS group.***A–D,* whole-cell currents in oocytes expressing PC2 F604P (*A*, *C*) or PC2 L677A N681A (*B*, *D*) were detected before and after 5 min incubation with BMTS (1 mM; *A*, *B*) or BMS (1 mM; *C*, *D*). Experiments were performed as described in Figure 3. *Left panels* Average I/V plots (mean ± SD) obtained in NaCl ØCa^2+^ØMg^2+^ solution before and after incubation with BMTS or BMS as indicated. *Right panels:* summary data from the experiments shown in the corresponding *left panels* depict maximal inward current values reached at −100 mV before and after incubation with the respective reagent. Lines connect data points obtained from the same oocyte. *p* values were calculated using paired two-tailed Student's *t* test. *E,* relative effect of BMTS or BMS on currents at −100 mV in % was calculated as described in Figure 4*C* using the current values shown in (*A*–*D*). *p* values were calculated using Kruskal–Wallis and Dunn's multiple comparisons test (N = 2, n = 5–12; N indicates the number of different batches of *Xenopus laevis* oocytes, and n indicates the number of individual oocytes analyzed per experimental group). PC2, polycystin-2; MTS, methanethiosulfonate; MS, methanesulfonate; BMTS, benzyl methanethiosulfonate; BMS, benzyl methanesulfonate.

To confirm that the functional MTS group is essential for mediating the inhibitory effect of lipophilic MTS reagents, we compared the effects of two structurally similar compounds, BMTS and benzyl methanesulfonate (BMS). The latter contains the methanesulfonate group with an oxygen atom instead of sulfur, which likely reduces its reactivity toward cysteines compared to the highly reactive MTS group of BMTS. As expected from previous experiments (Fig. 4, *A* and *C*), BMTS produced a strong inhibitory effect on PC2 F604P currents (Fig. 5, *A* and *E*). In contrast, the same concentration of BMS had no measurable effect on PC2 F604P (Fig. 5, *C* and *E*) and neither compound affected PC2 L677A N681A (Fig. 5, *B*, *D* and *E*). This finding suggests that the inhibitory effect of BMTS, and likely other lipophilic MTS reagents, is mediated by covalent cysteine modification of PC2 F604P.

**Figure 6:**
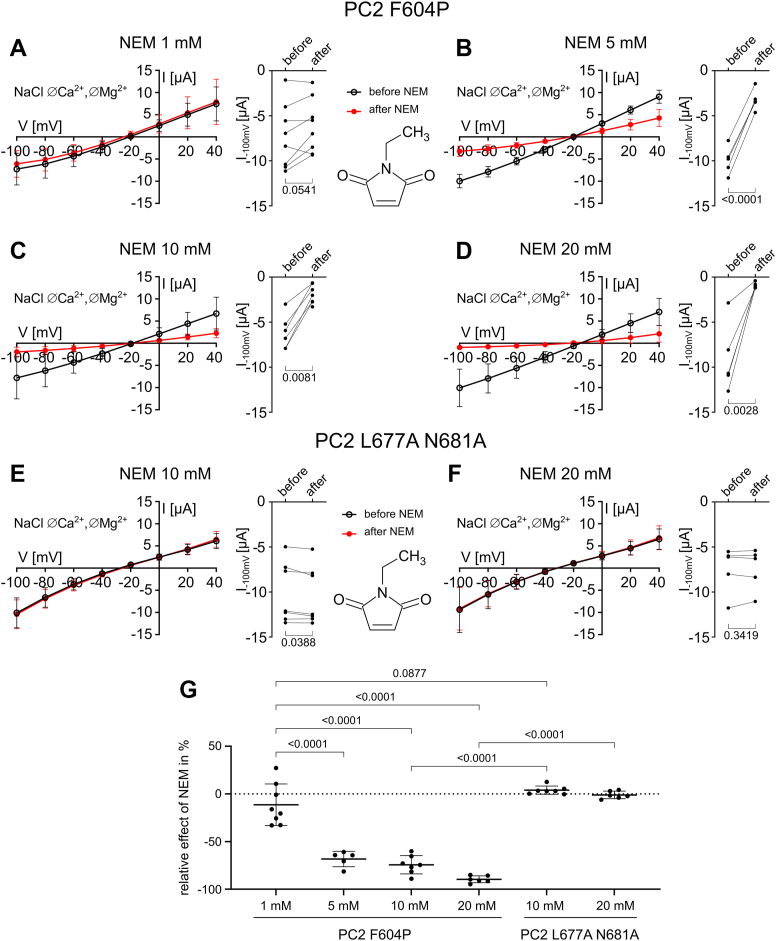
**The cysteine-reactive reagent NEM inhibited PC2 F604P but not PC2 L677A N681A.***A–F,* whole-cell currents in oocytes expressing PC2 F604P (*A*–*D*) or PC2 L677A N681A (*E*, *F*) were detected before and after 5 min incubation with NEM at the indicated concentrations. Experiments were performed as described in Figure 3. *Left panels:* average I/V plots (mean ± SD) obtained in NaCl ØCa^2+^ØMg^2+^ solution before and after incubation with NEM. *Right panels:* summary data from the experiments shown in the corresponding *left panels* depict maximal inward current values reached at −100 mV before and after incubation with NEM. Lines connect data points obtained from the same oocyte. *p* values were calculated using paired two-tailed Student's *t* test. *G,* relative effect of NEM on currents at −100 mV in % was calculated as described in Figure 4*C* using the current values shown in (*A*–*F*). *p* values were calculated using one-way ANOVA and Šídák's multiple comparisons test (N = 2, n = 5–8; N indicates the number of different batches of *Xenopus laevis* oocytes, and n indicates the number of individual oocytes analyzed per experimental group). PC2, polycystin-2.

We also tested whether PC2 F604P can be inhibited by NEM, a thiol-reactive agent that alkylates cysteine residues *via* a Michael-type addition. NEM is membrane-permeable and has been widely used for cysteine-specific labeling of transmembrane and intracellular proteins at concentrations from the supramicromolar to supramillimolar range (49, 50, 51, 52). Importantly, NEM inhibited PC2 F604P currents in a concentration-dependent manner (Fig. 6, *A–D* and *G*), but had no significant effect on PC2 L677A N681A even at the highest concentration tested (Fig. 6, *E*–*G*). Notably, achieving a near-complete inhibition of PC2 F604P required a 20-fold higher concentration of NEM than of MTS reagents, possibly reflecting the lower reactivity of NEM toward cysteines than the highly reactive MTS reagents (53).

To conclude, the ion channel function of PC2 F604P, but not of PC2 L677A N681A, can be inhibited by membrane-permeable cysteine-reactive compounds, most likely through covalent modification of functionally important cysteine residues. In contrast, charged MTS reagents had no significant inhibitory effect, probably because they did not reach the critical cysteine residues localized intracellularly.

### The closely related TRPML3 channel with a similar GOF mutation (A419P) was insensitive to MMTS application

**Figure 7:**
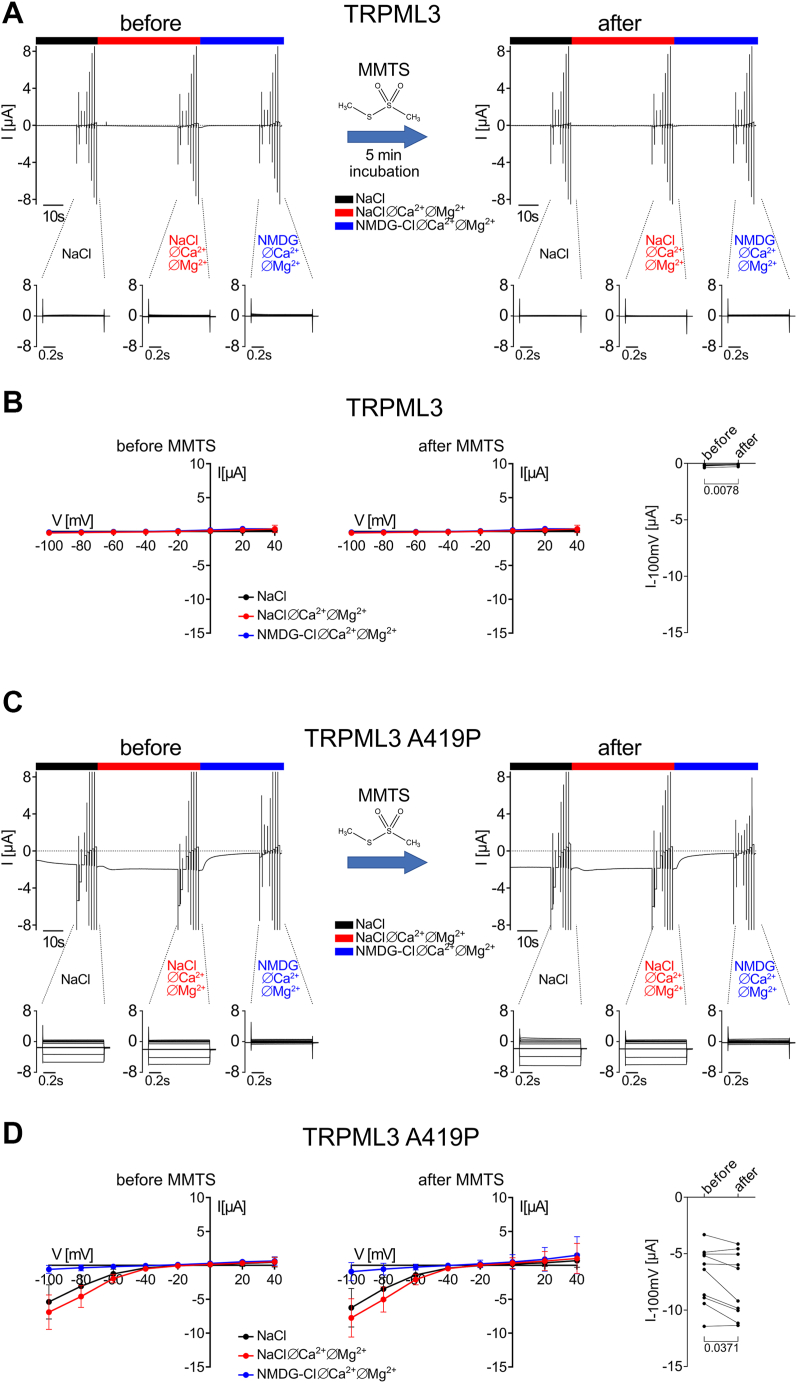
**A GOF mutant of TRPML3 (A419P) was not inhibited by MMTS application.***A* and *C*, Representative whole-cell current traces obtained in an oocyte expressing TRPML3 (*A*) or TRPML3 A419P (*C*) before (*left panels*) and after (*right panels*) 5 min incubation with MMTS (1 mM). Experiments were performed as described in Figure 3. *B* and *D, left and middle panels:* average I/V plots (mean ± SD) were constructed from similar experiments as shown in (*A*) and (*C*) as described in Figure 3. Data points represent average values from 8 to 10 oocytes (*B*: N = 2, n = 8; *D*: N = 2, n = 10; N indicates the number of different batches of *Xenopus laevis* oocytes, and n indicates the number of individual oocytes analyzed per experimental group). *Right panels:* summary data from the experiments shown in corresponding *left and middle panels* demonstrate maximal inward current values reached at −100 mV in NaCl ØCa^2+^ØMg^2+^ solution before and after MMTS application. *p* values were calculated using Wilcoxon matched pairs signed rank test. MMTS, methyl methanethiosulfonate; GOF, gain of function; TRPML, transient receptor potential mucolipin.

Ion channels belonging to the transient receptor potential mucolipin (TRPML) TRP subfamily are structurally similar to the TRPP (polycystin) subfamily members (54). Interestingly, the spontaneously occurring A419P mutation in TRPML3, which causes the varitint-waddler phenotype in mice (55, 56, 57), produces a GOF effect as demonstrated in electrophysiological studies (58). Like the F604P mutation in PC2, the A419P mutation is located within the S5 transmembrane domain of TRPML3 in an analogous position. Therefore, we hypothesized that MMTS might inhibit TRPML3 A419P in a similar manner as PC2 F604P. As shown in Figure 7, we could reproduce the reported GOF effect of the A419P mutation on TRPML3 ion channel function. Indeed, in oocytes expressing WT TRPML3 no measurable currents were detected in the presence or absence of divalent cations in the bath solution (Fig. 7, *A* and *B*). In contrast, in TRPML3 A419P expressing oocytes (Fig. 7, *C* and *D*) large inwardly rectifying whole-cell currents were observed in the presence of divalent cations in the bath solution consistent with previous reports (10, 58). These currents increased only slightly upon divalent cation removal and were largely reduced when extracellular Na^+^ was replaced by NMDG^+^. This confirmed that the inward currents mediated by TRPML3 A419P are mainly carried by Na^+^. Unlike polycystins, TRPML3 A419P was only marginally inhibited in the presence of divalent cations (Fig. 7, *C* and *D*). Importantly, MMTS had no significant effect on whole-cell currents from oocytes expressing WT TRPML3 (Fig. 7, *A* and *B*) or the TRPML3 GOF mutant (Fig. 7, *C* and *D*). To conclude, despite being closely related to PC2 F604P, the TRPML3 A419P mutant was insensitive to MMTS application. This indicates that MMTS-mediated inhibition is a rather specific feature of PC2 F604P.

### The free cysteine residue C593 localized in the S4-S5 linker is crucial for PC2 F604P ion channel function

**Figure 8:**
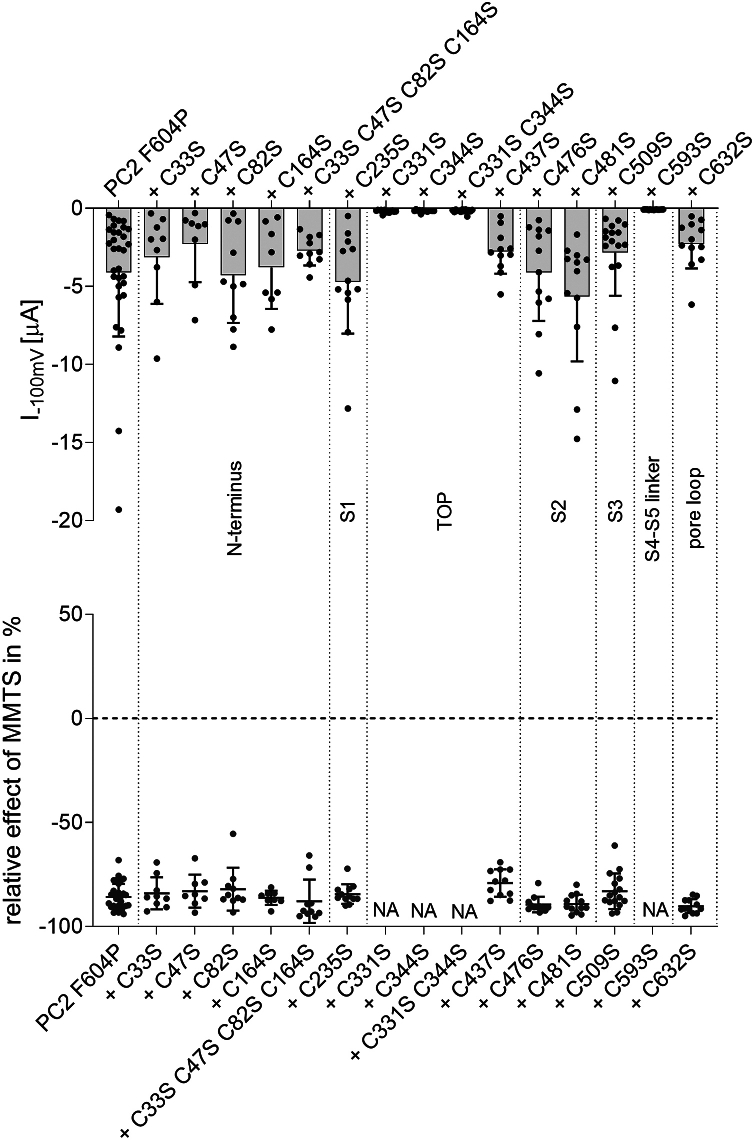
**Screening mutagenesis revealed C593 as a crucial cysteine residue for PC2 F604P ion channel function.** Whole-cell current values from oocytes expressing PC2 F604P without or with specified cysteine to serine substitutions were obtained essentially as described in Figure 3. *Upper panel*: maximal baseline inward currents in NaCl ØCa^2+^ØMg^2+^ solution at −100 mV (mean + SD and individual data points) are shown. *Lower panel*: relative inhibitory effect of MMTS in % was calculated as described in Figure 4. This calculation was not applicable (NA) to mutants that exhibited no detectable ion channel activity (N = 1–5, n = 8–31; N indicates the number of different batches of *Xenopus laevis* oocytes, and n indicates the number of individual oocytes analyzed per experimental group). PC2, polycystin-2; MMTS, methyl methanethiosulfonate.

To test whether individual cysteine residues are responsible for the inhibitory effect of MMTS on PC2 F604P, we performed a screening mutagenesis: each cysteine residue shown in Figure 1 was individually mutated to a serine. In addition, we generated constructs in which several cysteine residues were mutated simultaneously. The conservative serine substitution was chosen to minimize alterations in side chain properties. The resulting mutants were analyzed electrophysiologically using the established protocol as described in Figure 3. Ten out of the thirteen individual cysteine mutants were functional and produced robust baseline sodium inward currents under divalent cation-free conditions, which were similar to those obtained in oocytes expressing PC2 F604P without additional substitutions (Fig. 8, *upper panel*). Next, we investigated whether these functional mutants could be inhibited by MMTS. It has previously been reported that covalent modification of N-terminal cysteine residues by various thiol-reactive substances leads to activation of TRPA1 channels (59, 60). Additionally, an N-terminal cysteine (C38) in the closely related PKD2L1 channel has been identified as a palmitoylation site critical for channel function (61). Therefore, we particularly focused on the role of the N-terminal cysteine residues C33, C47, C82, and C164 in the MMTS-mediated inhibition. The individual cysteine-serine mutants (C33S, C47S, C82S, C164S), as well as the quadruple mutant (C33S C47S C82S C164S) were strongly inhibited by MMTS, similar to PC2 F604P with intact cysteines (Fig. 8, *lower panel*). This finding argues against a critical involvement of the N-terminal cysteine residues in the inhibitory effect of MMTS on PC2 F604P channel function. Importantly, the inhibitory effect of MMTS was also preserved in all other functional PC2 F604P constructs with cysteine to serine mutations (C235S, C437S, C476S, C481S, C509S, and C632S; Fig. 8, *lower panel*).

Interestingly, C331S and C344S were among the three mutants that failed to produce detectable currents in oocytes. As mentioned above, these cysteine residues are known to form a disulfide bond in the TOP domain of the channel, and the C331S mutation disrupts this disulfide bond (32). Notably, the C331S mutation is classified as “likely pathogenic” in the ADPKD variant database (https://pkdb.mayo.edu) and has been shown to cause a LOF effect on PC2 ion channel function in primary cilia (32). This aligns well with our findings. In addition, *Vien et al.* reported that PC2 currents could be restored by replacing the disulfide bond with a hydrogen bond formed by a double serine mutation (C331S C344S) (32). We were unable to reproduce this finding: in our hands the C331S C344S double mutant on the F604P background did not exhibit measurable currents (Fig. 8, *upper panel*). In contrast, the same cysteine-serine substitutions (C331S and C344S) introduced into the PC2 L677A N681A GOF construct did not significantly affect its currents (Fig. S4). Thus, the C331-C344 disulfide bond in the TOP domain appeared to be essential for the ion channel function of PC2 F604P but not for that of PC2 L677A N681A.

**Figure 9:**
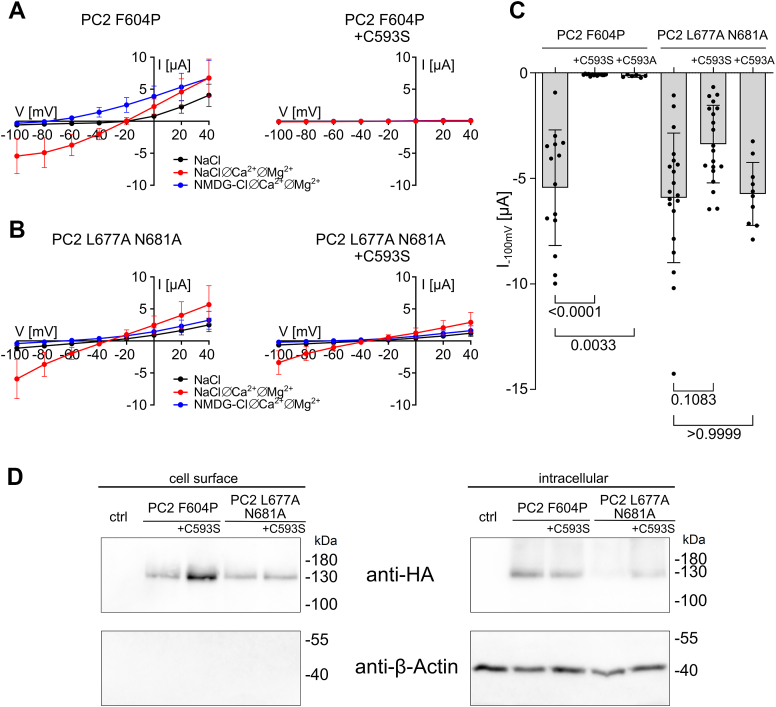
**Replacing C593 in the S4-S5 linker by serine inhibited PC2 F604P but not PC2 L677A N681A.***A* and *B,* average I/V plots (mean ± SD) in different bath solutions as indicated were obtained as described in Figure 3 in oocytes expressing PC2 F604P (*A*) or PC2 L677A N681A (*B*) without (*left panels*) or with (*right panels*) the additional C593S substitution. *C,* summary data from the same experiments shown in (*A*, *B*) and from similar experiments obtained in oocytes expressing the PC2 GOF constructs with the C593A substitution; for each experiment the maximal inward current reached at −100 mV in NaCl ØCa^2+^ØMg^2+^ bath solution (mean ± SD and individual data points are shown; N = 1–2, n = 6–20; N indicates the number of different batches of *Xenopus laevis* oocytes, and n indicates the number of individual oocytes analyzed per experimental group). *p* values were calculated using Kruskal–Wallis and Dunn's multiple comparisons test. *D,* western blot analysis of cell surface (*left panels*) and intracellular (*right panels*) expression of PC2 constructs in oocytes from the same batch. PC2 was detected using an antibody against the HA-tag attached to the N terminus of PC2. The separation of cell surface proteins from intracellular proteins was confirmed by reprobing the stripped western blots with an anti-β-actin antibody. Original uncropped images of the same western blots are shown in Figure S5. PC2, polycystin-2; GOF, gain of function; HA, hemagglutinin.

The third cysteine-to-serine mutation of PC2 F604P that failed to produce measurable currents was C593S, located in the S4-S5 linker. To rule out that the LOF effect of the C593S mutation was due to reduced cell surface expression of the channel, we performed a cell surface biotinylation assay. Interestingly, expression of PC2 F604P+C593S at the cell surface appeared to be enhanced compared to that of PC2 F604P without the C593S mutation (Fig. 9*D*; Fig. S5). We have no explanation for this enhanced surface expression, but it rules out the possibility that the LOF effect of the C593S mutation is caused by a lack of channel expression at the cell surface. Intriguingly, the functional effect of this serine substitution was similar to that of MMTS. Indeed, like MMTS, C593S specifically abolished the ion channel function of PC2 F604P, but not of PC2 L677A N681A (Fig. 9, *A*–*C*). In additional experiments, we also tested the effect of an alanine substitution of C593. Similar to C593S, C593A abolished the currents of PC2 F604P, but had no significant effect on PC2 L677A N681A (Fig. 9*C*).

To conclude, site-directed mutagenesis did not reveal a specific cysteine residue required for the inhibitory effect of MMTS on PC2 F604P. However, in addition to the well-established functional importance of the C331-C344 disulfide bond, it identified C593 in the S4-S5 linker as the only free cysteine residue essential for the ion channel function of PC2 F604P. Thus, it is conceivable that MMTS and other membrane-permeable cysteine-reactive compounds inhibit PC2 F604P through covalent modification of C593.

### Ion channel activity of PC2 F604P, but not PC2 L677A N681A, is inhibited by several structurally unrelated amphiphilic substances

Activity of various ion channels can be modulated by amphiphilic substances that probably integrate into the membrane and change its properties like thickness, curvature, and fluidity (62, 63). In addition to being thiol-reactive compounds, lipophilic MTS reagents as well as NEM interact with the plasma membrane. At millimolar concentrations, it is plausible that they alter the properties of the lipid bilayer and thereby lipid–protein interactions. Therefore, we tested whether structurally unrelated amphiphilic substances could reproduce the inhibitory effect of MTS reagents and NEM on PC2 F604P. The following substances were used because of their amphiphilic nature and their reported ability to modulate various ion channels (63, 64, 65, 66): chlorpromazine (CPZ), a Food and Drug Administration–approved antipsychotic drug; the widely used detergents Triton X-100 and n-dodecyl β-D-maltoside (DDM); and the vanilloid capsaicin.

**Figure 10:**
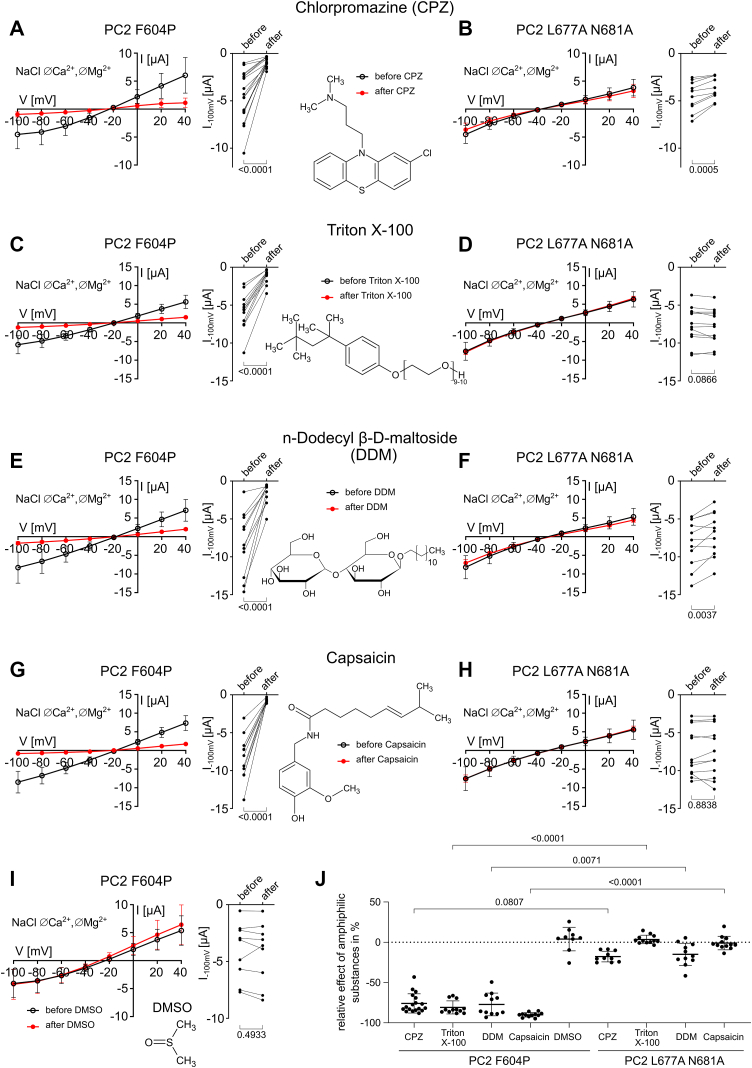
**Structurally unrelated amphiphilic compounds inhibited PC2 F604P but not PC2 L677A N681A.***A–I,* whole-cell currents in oocytes expressing PC2 F604P (*A, C, E, G*, and *I*) or PC2 L677A N681A (*B, D, F, H*) were detected before and after 5 min incubation with chlorpromazine (CPZ; 0,25 mM; *A, B*), Triton X-100 (0.1 mM; *C, D*), n-dodecyl β-D-maltoside (DDM; 0.1 mM; *E, F*), capsaicin (0.5 mM) or with DMSO used as vehicle (1:200; *I*) essentially as described in Figure 3. *Left panels:* average I/V plots (mean ± SD) in NaCl ØCa^2+^ØMg^2+^ bath solution before and after incubation with the indicated substance. *Right panels:* summary data from the experiments shown in corresponding *left panels* demonstrating maximal inward currents in individual oocytes reached at −100 mV in NaCl ØCa^2+^ØMg^2+^ bath solution before and after treatment with the indicated substance. *p* values were calculated using paired two-tailed Student's *t* test. *J,* relative effect of amphiphilic substances on currents at −100 mV in % was calculated as described in Figure 4 using the current values shown in (*A–I*). *p* values were calculated using Kruskal–Wallis and Dunn's multiple comparisons test (N = 1–2, n = 9–18; N indicates the number of different batches of *Xenopus laevis* oocytes, and n indicates the number of individual oocytes analyzed per experimental group). PC2, polycystin-2; DMSO, dimethyl sulfoxide.

To assess the effect of CPZ on PC2 F604P and PC2 L677A N681A, we incubated the oocytes in NaCl bath solution supplemented with 0.25 mM CPZ using our routine protocol as described in Figure 3. CPZ strongly inhibited PC2 F604P currents, reducing them by approximately 80% compared to the current level before CPZ treatment (Fig. 10, *A* and *J*). In contrast, CPZ produced almost no inhibition of PC2 L677A N681A-mediated currents (Fig. 10, *B* and *J*). Similar inhibitory effects on PC2 F604P were observed with Triton X-100 (0.1 mM; Fig. 10, *C* and *J*), DDM (0.1 mM; Fig. 10, *E* and *J*) and capsaicin (0.5 mM; Fig. 10, *G* and *J*), whereas PC2 L677A N681A was only marginally affected by the tested compounds (Fig. 10, *D*, *F*, *H* and *J*). We also applied dimethyl sulfoxide (DMSO; 1:200) as a vehicle control on PC2 F604P and observed no significant changes of the ion channel activity (Fig. 10, *I* and *J*).

To conclude, we demonstrated that several structurally unrelated amphiphilic compounds can inhibit PC2 F604P, but not PC2 L677A N681A. These findings suggest that PC2 F604P is particularly sensitive to membrane perturbations induced by membrane-active compounds. Therefore, it is conceivable that, in addition to covalently modifying C593, lipophilic MTS reagents and NEM may convey their inhibitory effect on PC2 F604P also by altering the channel's interaction with its lipid environment.

### Interaction of the S4-S5 linker of one PC2 subunit with the S6 transmembrane domain of the neighboring subunit appears to be critical for PC2 F604P ion channel function

**Figure 11:**
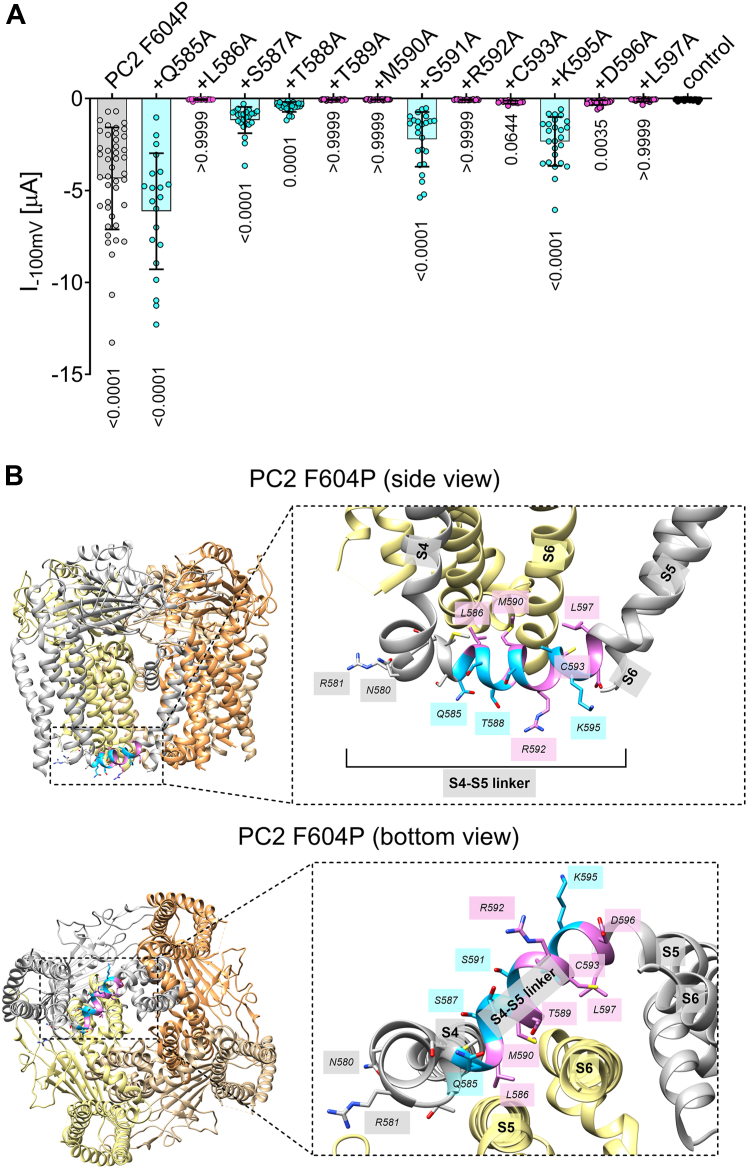
**The apparent molecular interaction of the S4-S5 linker of one PC2 subunit with the adjacent S6 transmembrane domain of the neighboring subunit is probably critical for PC2 F604P ion channel function.***A,* in experiments as described in Figure 3, whole-cell currents were measured in oocytes expressing PC2 F604P without or with additional alanine substitutions as specified. Maximal baseline inward currents in NaCl ØCa^2+^ØMg^2+^ solution at −100 mV (mean ± SD and individual data points) are shown. Alanine mutants of PC2 with (partially) preserved ion channel function are colored in *cyan*, mutants with a complete LOF effect are colored in *magenta*. *p* values for comparisons with the control group were calculated using Kruskal–Wallis and Dunn's multiple comparisons test (N = 2–5, n = 12–45; N indicates the number of different batches of *Xenopus laevis* oocytes, and n indicates the number of individual oocytes analyzed per experimental group). *B,* side and bottom view of human PC2 F604P in *ribbon representation* generated using atom coordinates from Protein Data Bank entry 6D1W (23). Individual PC2 subunits are depicted in different colors. The insets show a portion of PC2 F604P on an expanded scale. Residues within the S4-S5 linker are in *sticks representation* and colored in *cyan* or *magenta* according to the functional data shown in (*A*). PC2, polycystin-2; LOF, loss of function.

Building on the observation that the C593 residue within the S4-S5 linker is critical for the ion channel function of PC2 F604P, we focused on the functional role of the S4-S5 linker domain. We conducted an alanine screening, that is, the S4-S5 linker residues flanking the C593 residue (starting with Q585 and ending with L597) were individually substituted with alanine. Baseline sodium inward currents under divalent-free conditions were then assessed to identify substitutions that significantly affected the ion channel function of PC2 F604P. Our results revealed that the majority of alanine substitutions reduced or abolished PC2 F604P-mediated currents (Fig. 11*A*). Only one mutation (Q585A) had a fully preserved function, four mutants (S587A, T588A, S591A, and K595A) had a partially preserved function with significantly higher baseline currents (*p* ≤ 0.0001) than detected in control oocytes. The remaining alanine substitutions (L586A, T589A, M590A, R592A, C593A, D596A, and L597A) essentially abolished the ion channel function of PC2 F604P. Analysis of the available PC2 F604P structure revealed that the side chains in positions where alanine was tolerated (Q585, S587, T588, S591, A594, and K595) pointed away from the interface of two adjacent PC2 subunits (Fig. 11*B*). In contrast, the majority of residues critical for PC2 F604P ion channel function (L586, T589, M590, C593, and L597) had their side chains oriented toward the neighboring PC2 subunit and appeared to form close contacts especially with the S6 transmembrane domain (Fig. 11*B*).

These findings indicate that the intact interactions at the PC2 subunit interface between the S4-S5 linker of one subunit and the S6 transmembrane domain of the adjacent subunit are essential for PC2 F604P ion channel function.

### An ADPKD-associated mutation within the S4-S5 linker (N580K) disturbs ion channel function of PC2 F604P and PC2 L677A N681A

**Figure 12:**
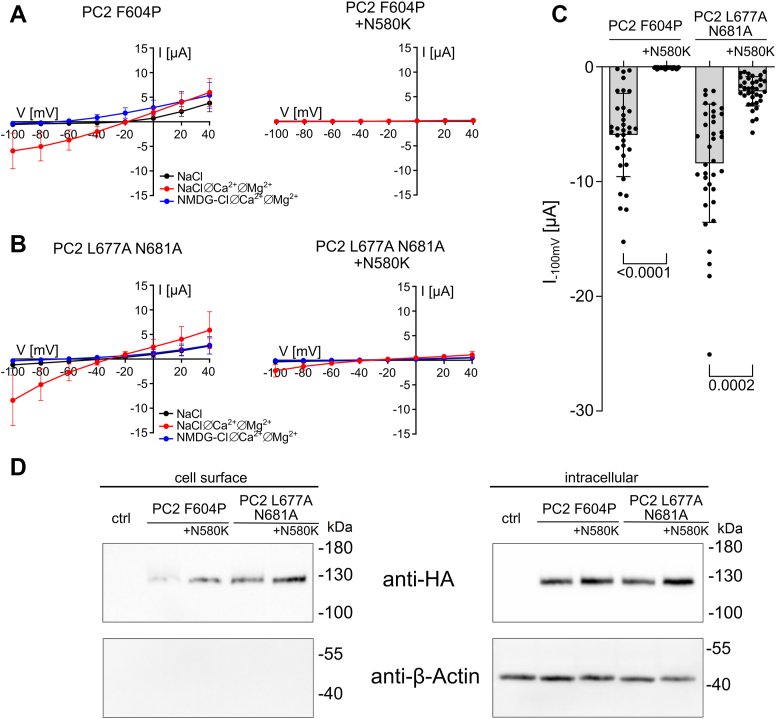
**ADPKD-associated mutation N580K in the S4-S5 linker disturbs the ion channel function of PC2 GOF constructs.***A* and *B,* average I/V plots (mean ± SD) in different solutions as indicated were obtained as described in Figure 3 in oocytes expressing PC2 F604P (*A*) or PC2 L677A N681A (*B*) without (*left panels*) or with (*right panels*) the additional ADPKD-associated mutation N580K. *C,* summary data from the same experiments shown in (*A, B*) demonstrating maximal inward currents reached at −100 mV in NaCl ØCa^2+^ØMg^2+^ bath solution (mean ± SD and individual data points are shown; N = 4, n = 35–36; N indicates the number of different batches of *Xenopus laevis* oocytes, and n indicates the number of individual oocytes analyzed per experimental group). *p* values were calculated using Kruskal–Wallis and Dunn’s multiple comparisons test. *D,* western blot analysis of cell surface (*left panels*) and intracellular (*right panels*) expression of PC2 constructs in oocytes from one batch performed as described in Figure 9. Similar results were obtained in three additional experiments shown in Figures S7 to S9. PC2, polycystin-2; ADPKD, autosomal-dominant polycystic kidney disease; GOF, gain of function.

According to the ADPKD variant database (https://pkdb.mayo.edu), there is one “likely pathogenic” missense mutation N580K located at the proximal end of the S4-S5 linker near its transition to the S4 transmembrane domain (Fig. 11*B*). Previously, it has been shown by the Yong Yu group that this mutation causes a LOF effect on PC2 F604P (43). We compared the functional impact of the N580K mutation on PC2 F604P *versus* PC2 L677A N681A. We confirmed the previously reported finding (43) that the N580K substitution completely abolished PC2 F604P ion channel function (Fig. 12, *A* and *C*). In contrast, the N580K mutation reduced PC2 L677A N681A currents by about 75% but with a significant residual function (Fig. 12, *B* and *C*). N580K mutation had no significant effect on the cell surface expression of both PC2 GOF constructs, as determined by western blot analysis (Fig. 12*D*, Figs. S6–S10). Thus, the N580K mutation is likely to inhibit ion channel function rather than channel expression at the cell surface.

To conclude, these findings suggest that the intact S4-S5 linker is crucial for the normal ion channel function of PC2. Moreover, they support the concept that disturbed PC2 ion channel function contributes to the pathophysiology of ADPKD.

## Discussion

In this study, we made the following key observations: (1) several membrane-permeable cysteine-reactive compounds, including MMTS and NEM, but not the positively-charged reagents MTSET and MTSEA, inhibited PC2 with the F604P GOF mutation almost completely and irreversibly; (2) in contrast, cysteine-reactive compounds had no effect on the PC2 L677A N681A GOF construct; (3) the closely related TRPML3 channel with the A419P GOF mutation was also insensitive to MMTS treatment; (4) screening mutagenesis revealed that, in addition to the C331 and C344 residues involved in disulfide bond formation, only one free cysteine residue, C593, within the intracellular S4-S5 linker plays a critical role in PC2 F604P channel function; (5) the C593S and C593A mutations abolished PC2 F604P but not PC2 L677A N681A channel function, mimicking the effect of cysteine-reactive compounds on these GOF channels; (6) similarly, several structurally unrelated amphiphilic substances caused near-complete and irreversible inhibition of PC2 F604P, whereas they had almost no effect on PC2 L677A N681A; (7) interactions between the S4-S5 linker of one PC2 subunit and the S6 transmembrane domain of the neighboring subunit probably play an important role in F604P-triggered PC2 opening; and (8) the ADPKD-associated mutation localized in the S4-S5-linker (N580K) essentially abolished PC2 F604P ion channel function and strongly reduced that of PC2 L677A N681A. Collectively, the data presented in the current study suggest that PC2 gating critically depends on the intact S4-S5 linker and the properties of the lipid bilayer.

**Figure 13:**
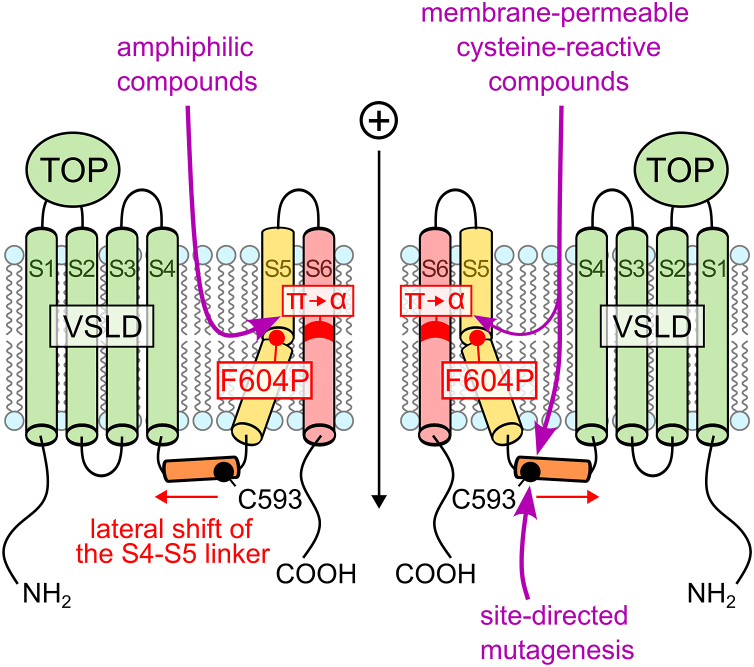
**Membrane-permeable cysteine-reactive compounds, amphiphilic substances, and mutations within the S4-S5 linker inhibit the PC2 F604P channel, probably by disturbing the π-to-α transition in the S6 domain.** The F604P mutation induces a conformational change in S5, which causes a lateral shift of the S4-S5 linker and triggers a π-to-α helix transition in S6 that opens the channel pore. Membrane-permeable cysteine-reactive compounds may covalently modify C593 in the S4-S5 linker, altering its conformation and disrupting its interactions with S6, thereby preventing the π-to-α transition. Mutations within the S4-S5 linker likely act in a similar manner. Alternatively, membrane-permeable cysteine-reactive compounds, like amphiphilic substances, may change membrane properties and alter lipid–protein interactions, also interfering with the π-to-α switch in S6. TOP, tetragonal opening of polycystins; VSLD, voltage sensor–like domain; S1-S6, transmembrane domains 1 to 6; π, π helix; α, α helix; PC2, polycystin-2.

Our observation that PC2 F604P can be inhibited by membrane-permeable lipophilic thiol-reactive MTS reagents, but not by the membrane-impermeable positively charged MTSET and MTSEA, suggested that the inhibitory effect may be mediated by covalent modification of intracellular free cysteine residues. This hypothesis was further supported by the observation that an intact MTS group in BMTS was required to produce PC2 F604P inhibition. Indeed, BMS, which differs from BMTS only by an oxygen-for-sulfur substitution within the MTS group, failed to produce the inhibitory effect. Furthermore, another membrane-permeable cysteine-reactive compound, NEM, also inhibited PC2 F604P to the same extent as the lipophilic MTS reagents, albeit at higher concentrations. However, certain observations do not fully align with the proposed mechanism of inhibition. While quenching MMTS with DTT effectively prevented inhibition of PC2 F604P, incubating oocytes with DTT after MMTS treatment did not restore channel activity. This seemingly negative finding should, however, be interpreted with caution. Indeed, the lack of a rescuing effect of DTT may be due to inefficient removal of methyl adducts from cysteine residues under our experimental conditions. Alternatively, it is conceivable that covalent modification of PC2 F604P by MMTS shifts the channel into a stable inactive state that persists even after adduct removal. Importantly, our cysteine-to-serine screening mutagenesis failed to identify a specific cysteine residue responsible for the inhibitory effect of MMTS on PC2 F604P. Nevertheless, this analysis revealed a critical role of the free cysteine residue C593, localized within the intracellular S4-S5 linker, in PC2 F604P channel function. Moreover, the C593S and C593A mutations completely abolished the function of PC2 F604P but had little effect on PC2 L677A N681A, mimicking the effect of lipophilic cysteine-reactive compounds on these GOF constructs. The LOF effect of the C593S mutation makes it impossible to directly assess the role of this cysteine residue in mediating the inhibitory action of MMTS on PC2 F604P. Nevertheless, it is tempting to speculate that MMTS and other membrane-permeable cysteine-reactive compounds tested in this study inhibited PC2 F604P through covalent modification of C593 (Fig. 13). Functional effects of covalent modification of specific residues have previously been reported for TRP channels. In particular, it has been demonstrated that allicin and several other naturally occurring thiol-reactive substances can activate TRPA1 and TRPV1, probably through covalent modification of N-terminal cysteine and lysine residues (59, 60, 67, 68). Notably, oxidative modification of free cysteine residues, for example by ROS, is a common posttranslational modification mechanism in proteins (69). Thus, a similar mechanism involving the C593 residue may also be relevant for PC2 gating.

It should be noted that all cysteine-reactive compounds that inhibited PC2 F604P were membrane-permeable and were applied at high concentrations. Therefore, it is conceivable that these reagents, at least in part, exerted their inhibitory effects on PC2 F604P by altering the properties of the lipid bilayer, thereby affecting lipid–protein interactions and disturbing the F604P-induced channel opening (Fig. 13). Consistent with this interpretation, we observed that ion channel function of PC2 F604P was strongly and irreversibly inhibited by several structurally unrelated amphiphilic substances. This finding suggests that F604P-mediated gating of PC2 is highly sensitive to membrane perturbations likely induced by these membrane-active compounds (Fig. 13). Modulation of ion channel function by amphiphilic substances and membrane alterations (*i.e.*, *via* cholesterol depletion) is a well-established phenomenon demonstrated by different research groups including our own (63, 70, 71, 72, 73, 74, 75, 76). The proposed sensitivity of PC2 to the lipid environment is also supported by structural analysis. Indeed, structural analysis of PC2 revealed the presence of specific binding sites for phosphatidylinositol bisphosphate and cholesterol within its transmembrane domains (22, 24, 25). Moreover, *Ha et al.* recently demonstrated that the PC1-PC2 heterotetrameric channels can be activated by cilia-enriched 7β,27-dihydroxycholesterol *via* a specific binding pocket within PC2 (29). This aligns well with the concept that ADPKD is caused by a disturbed function of polycystins in the primary cilium (11), which probably comprises a unique lipid environment distinct from that of the plasma membrane (77).

We demonstrated that membrane-permeable cysteine-reactive compounds and amphiphilic substances specifically inhibited PC2 F604P but not PC2 L677A N681A. In addition, MMTS failed to affect the function of the closely related channel TRPML3 with the analogous A419P GOF mutation. This observation suggests that PC2 F604P, but not PC2 L677A N681A or TRPML3 A419P, possesses a specific feature that makes it susceptible to cysteine modifications and alterations of membrane properties. Importantly, activation of PC2 F604P involves the π-to-α transition in the S6 domain (23; Fig. 13), whereas PC2 L677A N681A is probably activated by removal of the lower gate constriction without a conformational change in the S6 domain (34). There is no structure of TRPML3 A419P available to date. However, structural analysis of TRPML3 activation by ML-SA1—a mucolipin synthetic agonist 1 which binds close to the A419 position—revealed that the π-helix in the S6 domain remained intact in the presence of the agonist with the channel in an open state (78). Thus, it is plausible that the GOF effect of the A419P mutation in TRPML3 does not involve a π-to-α switch, which may explain the lack of MMTS sensitivity of this channel. One could argue that in PC2 F604P the GOF effect of the proline substitution (or the π-to-α transition in the S6 domain itself) is intrinsically unstable and can therefore be readily converted into a more energetically favorable closed state, for example, by cysteine modifications or by alterations of membrane properties resulting from the integration of amphiphilic substances into the plasma membrane at the lipid-protein interface.

Our findings underscore the functional importance of the S4-S5 linker and particularly its interactions with the S6 domain of the neighboring subunit for ion channel function of PC2 F604P. Introducing subtle changes to this domain by individual side chain substitutions, even by the conservative C593S or C593A mutations, abolished PC2 F604P activity. Our observations correspond well with the results of the structural analysis of PC2 F604P. Indeed, it has been demonstrated that the F604P-induced PC2 opening involves a significant lateral shift of the S4-S5 linker, which is probably required for the π-to-α transition in the S6 domain and the consequent opening of the channel's lower gate (23; Fig. 13). Mutations within the S4-S5 linker may disturb interactions at the PC2 subunit interface, thereby preventing the π-to-α switch in the S6 domain and activation of the channel. Given its essential functional role in PC2 F604P, the S4-S5 linker is likely involved, directly or indirectly, in mediating the channel's sensitivity to cysteine-reactive and amphiphilic substances.

Like in related voltage-gated ion channels, the S4-S5 linker is a critical structural and functional element in TRP channels. The S4-S5 linker connects the VSLD with the PD. Conformational changes elicited, for example, by ligand binding to the VSLD, are transduced through movements of the S4-S5 linker to the PD to drive gating (31, 79, 80, 81). Furthermore, the S4-S5 linker can directly participate in the formation of ligand binding sites, like the vanilloid-binding pocket in TRPV channels (31, 82, 83). Interestingly, in PC2 a putative phosphatidylinositol bisphosphate/detergent binding site has been proposed, which corresponds to the vanilloid-binding site in TRPV1 (24). Thus, PIPs, amphiphilic, or lipophilic substances may bind to PC2 in the vicinity of the S4-S5 linker and possibly modulate its function.

Notably, the S4-S5 linker appears to be a hot-spot for mutations in several TRP channels, including TRPV3 (congenital Olmsted syndrome), TRPV4 (skeletal dysplasia, motor/sensory neuropathies), TRPA1 (familial episodic pain syndrome), and TRPML1 (mucolipidosis type IV) (84). These disease-causing mutations dramatically alter the gating properties of the respective channels, highlighting the functional significance of the S4-S5 linker. Importantly, the ADPKD-associated mutation N580K investigated in the present study completely abolished and significantly reduced the currents mediated by PC2 F604P and PC2 L677A N681A, respectively. The N580K mutation introduces a positively charged lysine, which is probably incompatible with the neighboring positively charged asparagine residue R581. This may cause a substantial conformational change within the S4-S5 linker and adjacent pore forming domains blocking the ion permeation pathway.

To conclude, this study provides new insights into the ion channel function of PC2 at the molecular level. In particular, it suggests the critical role of the membrane environment and the intact S4-S5 linker in PC2 gating. In addition, our findings contribute to a better understanding of how ADPKD-associated mutations may affect PC2 ion channel function.

## Experimental procedures

### Chemicals

MMTS, NEM, Triton X-100, capsaicin, and DTT were obtained from Sigma-Aldrich. BMTS, ethyl methanethiosulfonate, and propyl methanethiosulfonate were obtained from Toronto Research Chemicals. MTSET and MTSEA were purchased from Biotium. BMS was purchased from Cymit Química S.L. DDM was obtained from Thermo Fisher Scientific. CPZ was obtained from ChemCruz. Stock solutions in dimethyl sulfoxide were prepared for CPZ (50 mM), BMTS (500 mM), BMS (500 mM), and capsaicin (200 mM). Other compounds were directly dissolved in the incubation solution to the final concentrations as indicated in the text and figure legends. To prevent degradation of the MTS reagents, solutions were held on ice before application.

### Expression constructs

Full-length complementary DNA encoding human PC2 was kindly provided by R. Witzgall. For optimal heterologous expression in *Xenopus* *laevis* oocytes, PC2 was subcloned into the pTLN vector (85). Different point mutations, as well as an N-terminal hemagglutinin-tag (YPYDVPDYA), were introduced by site-directed mutagenesis using QuikChange Lightning Kit (Agilent). Sequences of all constructs were verified by the LGC Genomics sequencing service. Plasmids were linearized using Mlu I (Mlu I-HF, New England BioLabs) and used as templates for circular RNA (cRNA) synthesis using SP6 RNA polymerase (mMESSAGE mMACHINE SP6 Kit, Ambion).

### Isolation of oocytes and TEVC experiments

Isolation of *Xenopus* *laevis* oocytes and TEVC experiments were performed essentially as described previously (34, 48, 86). Ovarian lobes were excised by partial ovariectomy under anesthesia with Tricaine 0.2%, in accordance with the principles of German legislation, with approval by the animal welfare officer for the University of Erlangen-Nürnberg (FAU), and under the governance of the state veterinary health inspectorate. Stage V to VI oocytes were isolated from the ovarian lobes by enzymatic digestion and injected with 10 to 15 ng of cRNA encoding a corresponding PC2 construct. An antisense phosphorothioate DNA oligonucleotide (5-GCT TTA GTA ATT CCC ATC CTG CCA TGT TTC-3; 3 ng) was coinjected into the oocytes together with the cRNA to suppress the expression and possible interference of endogenous connexin 38 (Cx38) hemichannels (34, 48, 86, 87, 88). Control oocytes were injected with 3 ng of the anti-Cx38 oligonucleotide only. After injection, oocytes were incubated in ND9 solution (composition in mM: 87 N-methyl-D-glutamine-Cl (NMDG-Cl), 9 NaCl, 2 KCl, 1.8 CaCl_2_, 1 MgCl_2_, 5 Hepes, pH 7.4 adjusted with Tris) supplemented with sodium penicillin (100 units/ml) and streptomycin sulphate (100 μg/ml) at 19 °C for 48 h. For TEVC experiments, single oocytes were placed in a flow chamber and were superfused with a corresponding bath solution at a flow rate of approximately 10 ml/min. A modified ND96 solution was used as a standard NaCl bath solution (composition in mM: 96 NaCl, 4 KCl, 1 CaCl_2_, 1 MgCl_2_, 10 Hepes, pH 7.4 adjusted with Tris). To obtain a NaCl bath solution nominally free of divalent cations (NaCl ØCa^2+^ØMg^2+^), CaCl_2_, and MgCl_2_ were excluded from the ND96 solution. Subsequent replacement of 95 mM NaCl with the same concentration of NMDG-Cl resulted in an NMDG-Cl ØCa^2+^ØMg^2+^ bath solution. Bath solution exchanges with a gravity-fed system were controlled by a magnetic valve system (ALA VM8; ALA Scientific Instruments). Measurements were conducted at room temperature using a continuous holding potential of −60 mV. At the end of each solution application, a voltage step protocol was performed as described in the legend of Figure 3.

### Detection of PC2 at the cell surface and western blot analysis

To assess cell surface expression of PC2, a biotinylation approach and western blot analysis were performed essentially as described previously (34, 86, 89). In brief, oocytes were incubated with EZ-Link sulfo-NHS-SS-Biotin (Thermo Fisher Scientific) and lysed mechanically using 27G syringe needles. Biotinylated cell surface proteins were separated from intracellular proteins using NeutrAvidin Agarose beads (Pierce). After separation by SDS-PAGE, the proteins were transferred to a polyvinylidene difluoride membrane *via* semidry electroblotting. Hemagglutinin-tagged PC2 was then detected using a monoclonal rat anti-HA antibody (Roche Diagnostics) at a dilution of 1:1000 and a secondary horseradish peroxidase-conjugated goat-anti-rat antibody (Jackson Immunoresearch) at a dilution of 1:10,000. Subsequently western blots were stripped and restained for β-actin to confirm the separation of cell surface proteins from intracellular proteins. β-Actin was detected using a polyclonal rabbit anti-β-actin antibody (Sigma-Aldrich) at a dilution of 1:5000 and a secondary peroxidase-conjugated goat-anti-rabbit antibody (Invitrogen) at a dilution of 1:50,000. Blots were developed using SuperSignal West Femto (Thermo Fisher Scientific) and signals were captured with a Fusion SL imager (Vilber Lourmat). ATX Ponceau S (Fluka) membrane staining was used to control for protein loading.

### Data analysis and statistical tests

Analysis of the TEVC experiments was performed using the “Nest-o-Patch” (http://sourceforge.net/projects/nestopatch) program written by Dr V. Nesterov (Friedrich-Alexander-Universität Erlangen-Nürnberg, Institute of Cellular and Molecular Physiology). Densitometric analysis of western blots was performed using ImageJ (Rasband, W.S., ImageJ, U. S. National Institutes of Health, https://imagej.net/ij/, 1997–2018). Data are represented as mean ± SD. N indicates the number of different oocyte preparations; n indicates the number of individual oocytes per experimental group. Statistical evaluation was performed using an appropriate statistical test as indicated in figure legends using GraphPad Prism version 10 (GraphPad Software Inc; https://www.graphpad.com/). The normal distribution of data was assessed using the D'Agostino-Pearson omnibus test. Graphical representations were created using GraphPad Prism version 10. Structure visualizations were prepared using UCSF Chimera developed by the Resource for Biocomputing, Visualization, and Informatics at the University of California, with support from the National Institutes of Health P41-GM103311 (90).

## Data availability

All data are contained within the article and the supporting information.

## Supporting information

This article contains supporting information.

## Conflict of interest

The authors declare that they have no conflicts of interest with the contents of this article.
