# Supplementary material for: Differential modulation of polycystin-2 gain-of-function channels by cysteine-reactive compounds, amphiphilic substances, and S4-S5 linker mutations
Source: J Biol Chem. 2025 Sep 24;301(11):110766. doi: 10.1016/j.jbc.2025.110766 (PMC12597267; doi:10.1016/j.jbc.2025.110766)
Supplement: Supporting information [file mmc1.pdf]

Differential modulation of polycystin-2 gain-of-function channels by cysteine-reactive compounds, amphiphilic substances and S4-S5 linker mutations

– Supporting Information –

Linda Geiges, Tobias Staudner, Juthamas Khamseekaew, Christoph Korbmacher and Alexandr V. Ilyaskin

Table of Contents

Figure S1 MMTS incubation did not significantly alter currents in control oocytes.

Figure S2 Application of an MMTS-containing solution supplemented with the reducing agent DTT did not change PC2 F604P-mediated currents.

Figure S3 Treatment with DTT after MMTS did not restore PC2 F604P ion channel function.

Figure S4 Disruption of the cysteine bridge in the TOP domain did not significantly affect PC2 L677A N681A ion channel function.

Figure S5 Original uncropped images of the same western blots as shown in Figure 9d.

Figure S6 Western blot analysis of PC2 constructs with the N580K mutation (oocyte batch 1).

Figure S7 Western blot analysis of PC2 constructs with the N580K mutation (oocyte batch 2).

Figure S8 Western blot analysis of PC2 constructs with the N580K mutation (oocyte batch 3).

Figure S9 Western blot analysis of PC2 constructs with the N580K mutation (oocyte batch 4).

Figure S10 The N530K mutation did not significantly affect PC2 expression at the cell surface.

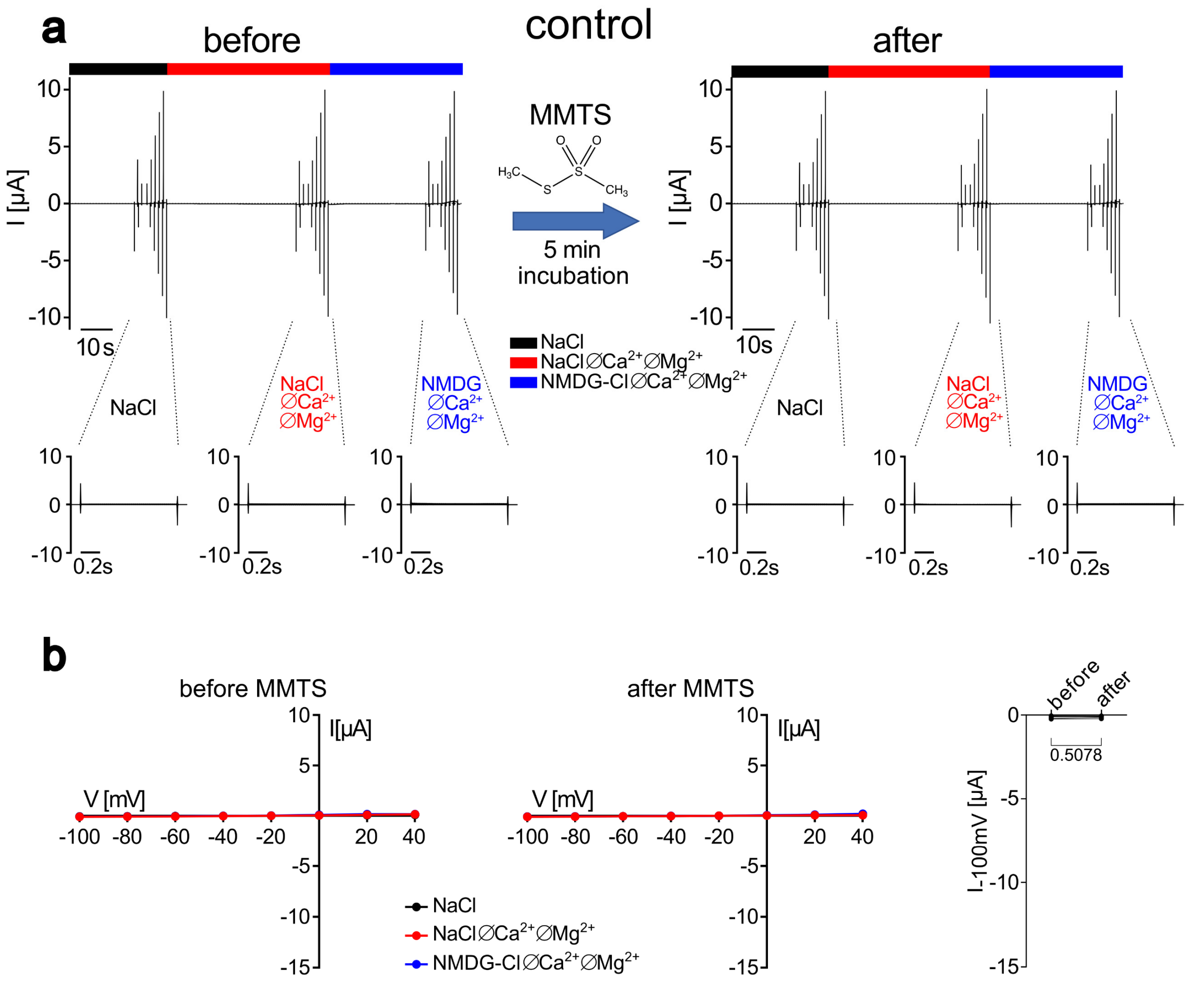

Fig. S1 MMTS incubation did not significantly alter currents in control oocytes. a, Representative whole-cell current traces obtained in a control oocyte before (*left panel*) and after (*right panel*) 5 min incubation in ND9 solution supplemented with MMTS (1 mM). Application of different bath solutions during the current measurement is indicated by bars. Experiments were performed using a similar protocol as described in Figure 3. **b**, *left and middle panels* Average I/V plots (mean ± SD) were constructed from similar experiments as shown in A using the mean current values measured during the last 300 ms of the voltage pulses. Data points represent average values from 10 oocytes (N=2, n=10; N indicates the number of different batches of *Xenopus laevis* oocytes, and n indicates the number of individual oocytes analyzed per experimental group). *Right panels* Summary data from the same experiments shown in *left* and *middle* panels demonstrate maximal inward current values reached during application of hyperpolarizing pulses of −100 mV in NaCl ØCa^{2+}ØMg^{2+} solution before and after MMTS application. *P-values* were calculated using Wilcoxon matched-pairs signed rank test.

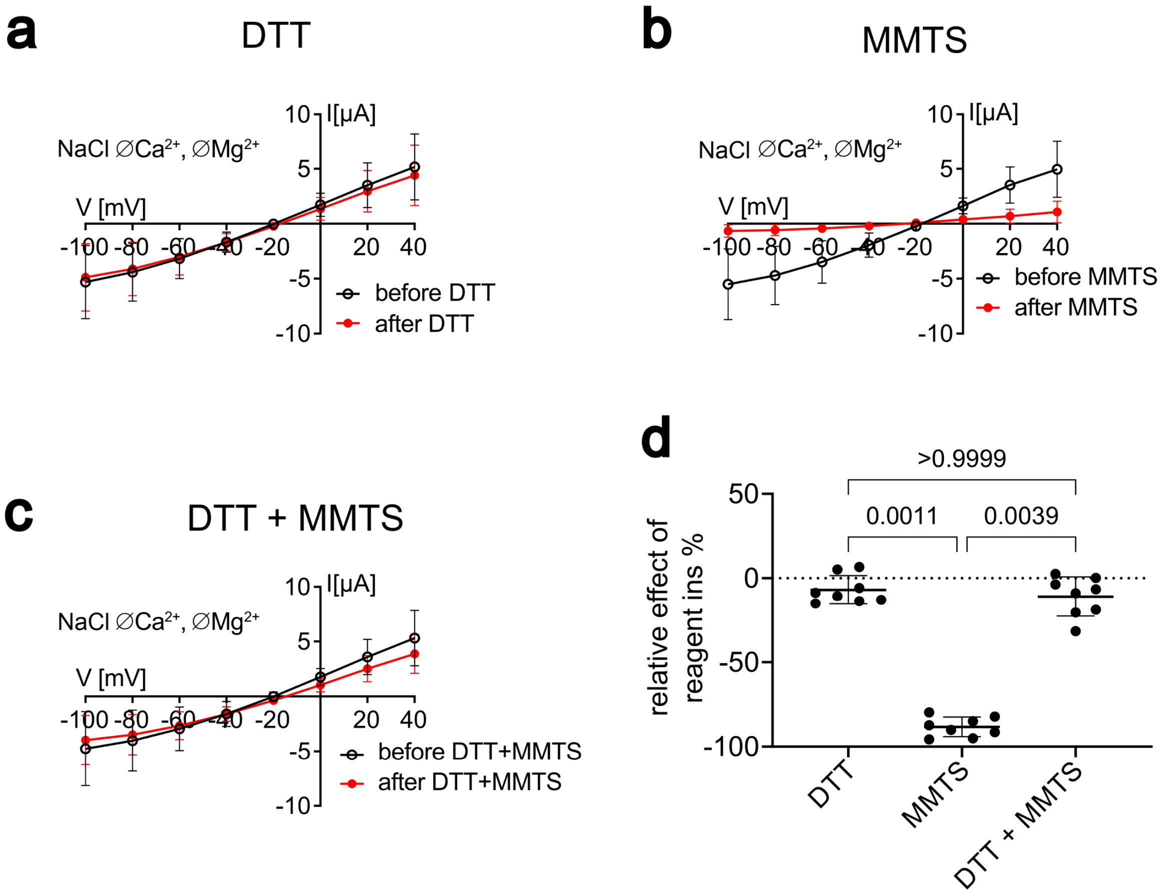

Fig. S2 Application of an MMTS-containing solution supplemented with the reducing agent DTT did not change PC2 F604P-mediated currents. a, Average I/V plots (mean ± SD) obtained in NaCl ØCa^{2+}ØMg^{2+} bath solution from oocytes expressing PC2 F604P. In each individual oocyte currents were measured before and after 5 min incubation in NaCl bath solution containing DTT (30 mM), MMTS (1 mM), or DTT (30 mM) + MMTS (1 mM) as indicated. Experiments were performed essentially as described in Figure 3. **b**, Relative effect of different incubation solutions in % calculated using the current values shown in **a** as described in Figure 4**c**. *P-values* were calculated using Kruskal-Wallis and Dunn's multiple comparisons test (N=2, n=8; N indicates the number of different batches of *Xenopus laevis* oocytes, and n indicates the number of individual oocytes analyzed per experimental group).

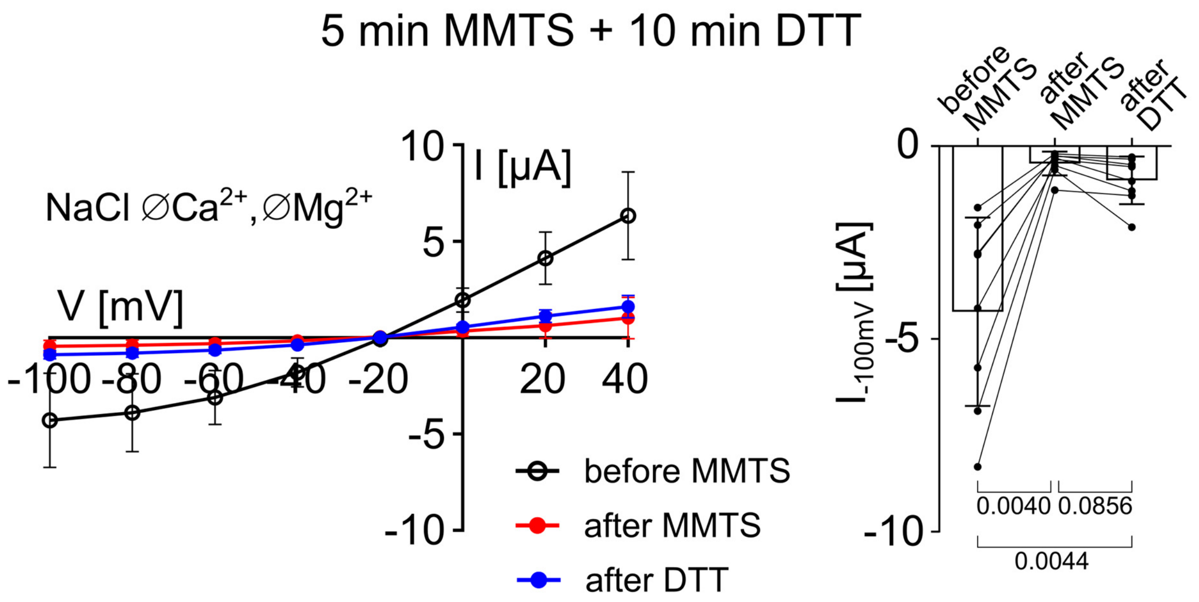

Fig. S3 Treatment with DTT after MMTS did not restore PC2 F604P ion channel function. *Left panel* Average I/V plots (mean ± SD) obtained in NaCl ØCa^{2+}ØMg^{2+} bath solution from oocytes expressing PC2 F604P. In each individual oocyte currents were measured three times: before application of MMTS, after 5 min incubation with MMTS (1 mM), and finally after 10 min incubation with DTT (30 mM). MMTS and DTT were dissolved in ND9 solution. Experiments were performed essentially as described in Figure 3. *Right panel* Summary data from the same experiments shown in *left panel* demonstrate maximal inward current values reached during application of hyperpolarizing pulses of −100 mV in NaCl ØCa^{2+}ØMg^{2+} solution before MMTS treatment, after MMTS treatment, and after DTT treatment. *P-values* were calculated using repeated measures one-way ANOVA and Tukey's multiple comparisons test (mean ± SD and individual data points are shown; N=2, n=8; N indicates the number of different batches of *Xenopus laevis* oocytes, and n indicates the number of individual oocytes analyzed per experimental group).

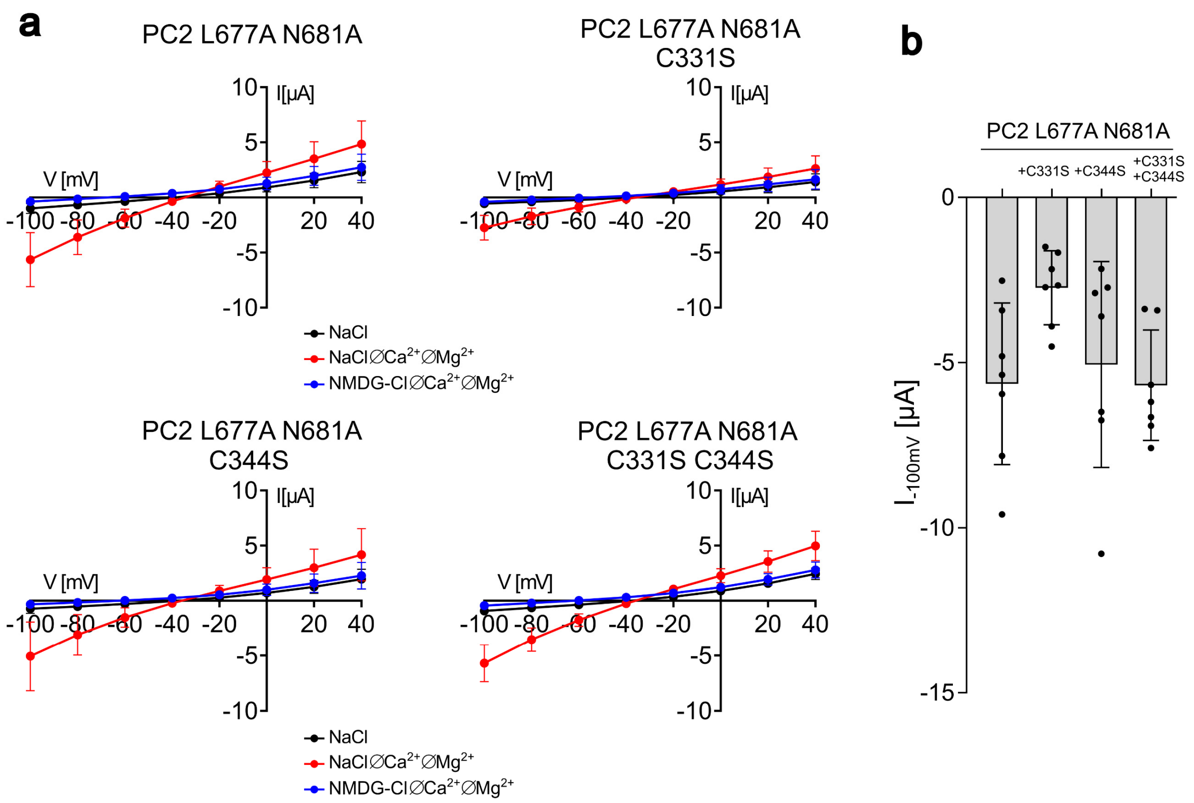

Fig. S4 Disruption of the cysteine bridge in the TOP domain did not significantly affect PC2 L677A N681A ion channel function

a Average I/V plots (mean ± SD) in different solutions as indicated were obtained as described in Figure 3 in oocytes expressing PC2 L677A N681A without or with additional cysteine to serine substitutions. **b** Summary data from the same experiments as shown in **a** depicting maximal inward currents reached at -100 mV in NaCl ØCa^{2+}ØMg^{2+} bath solution (mean ± SD and individual data points are shown; N=1, n=7; N indicates the number of different batches of *Xenopus laevis* oocytes, and n indicates the number of individual oocytes analyzed per experimental group).

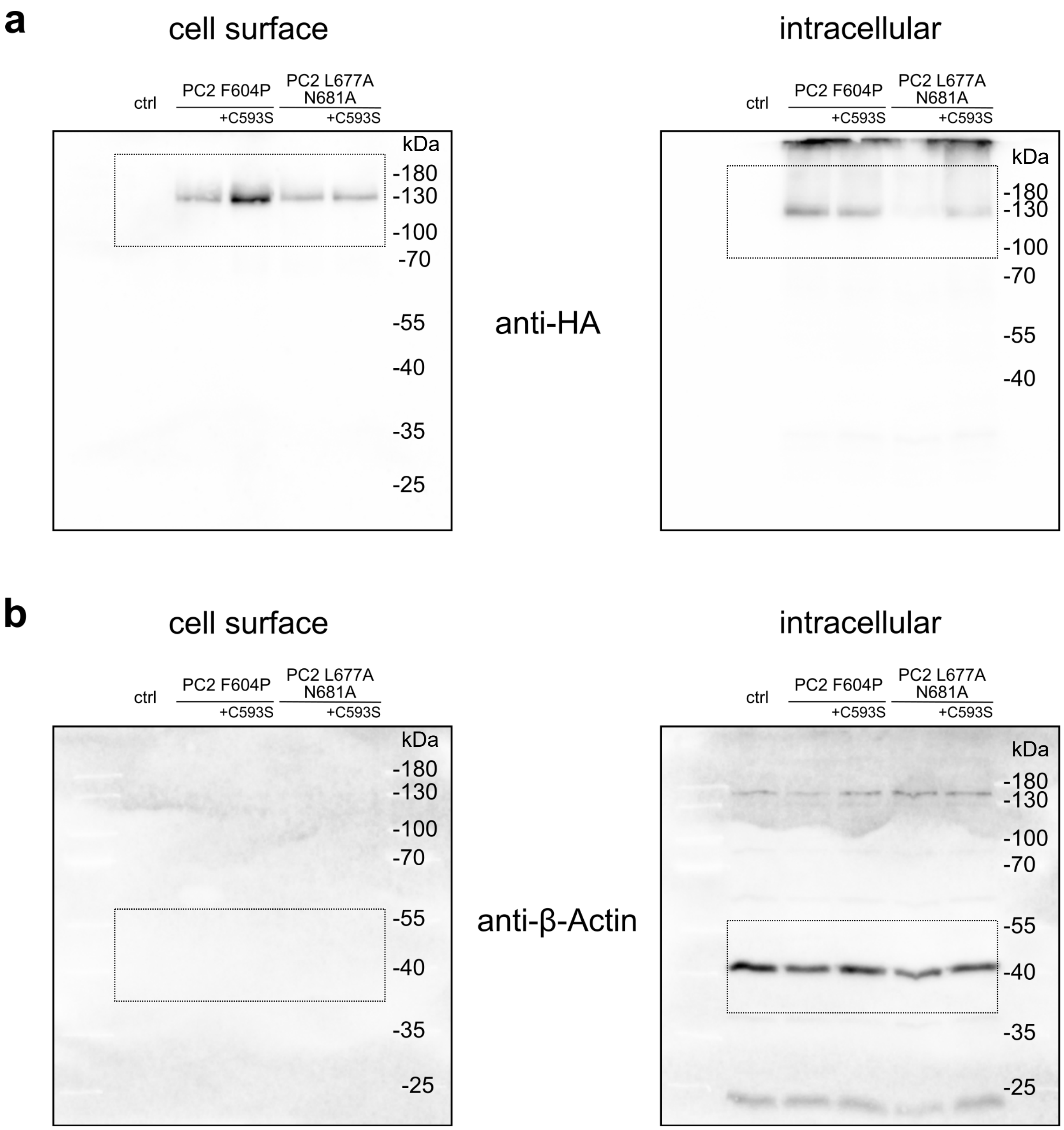

Fig. S5 Original uncropped images of the same western blots as shown in Figure 9d. a, Cell surface (*left panel*) and intracellular (*right panel*) expression of PC2 constructs was detected using an antibody against the N-terminal HA-tag. **b**, To confirm separation of cell surface proteins from intracellular proteins, the membranes were stripped and re-probed using an anti-β-actin antibody. Parts of blots shown in Fig. 9**d** are framed by dotted rectangles.

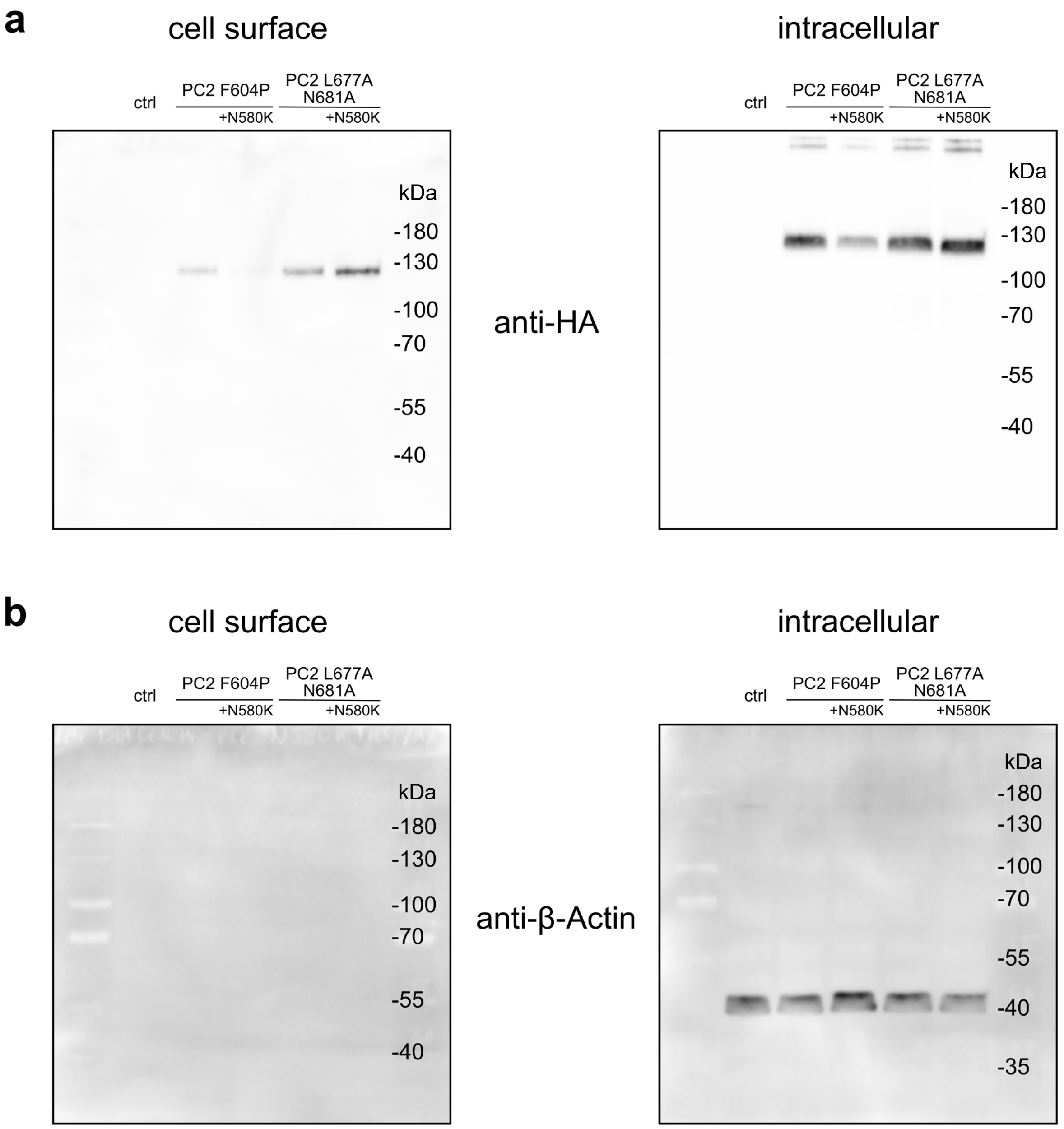

Fig. S6 Western blot analysis of PC2 constructs with the N580K mutation (oocyte batch 1) Cell surface (*left panel*) and intracellular (*right panel*) expression of PC2 mutants was detected using an antibody against the N-terminal HA-tag. **b**, To confirm separation of cell surface proteins from intracellular proteins, the membranes were stripped and re-probed using an anti-β-actin antibody.

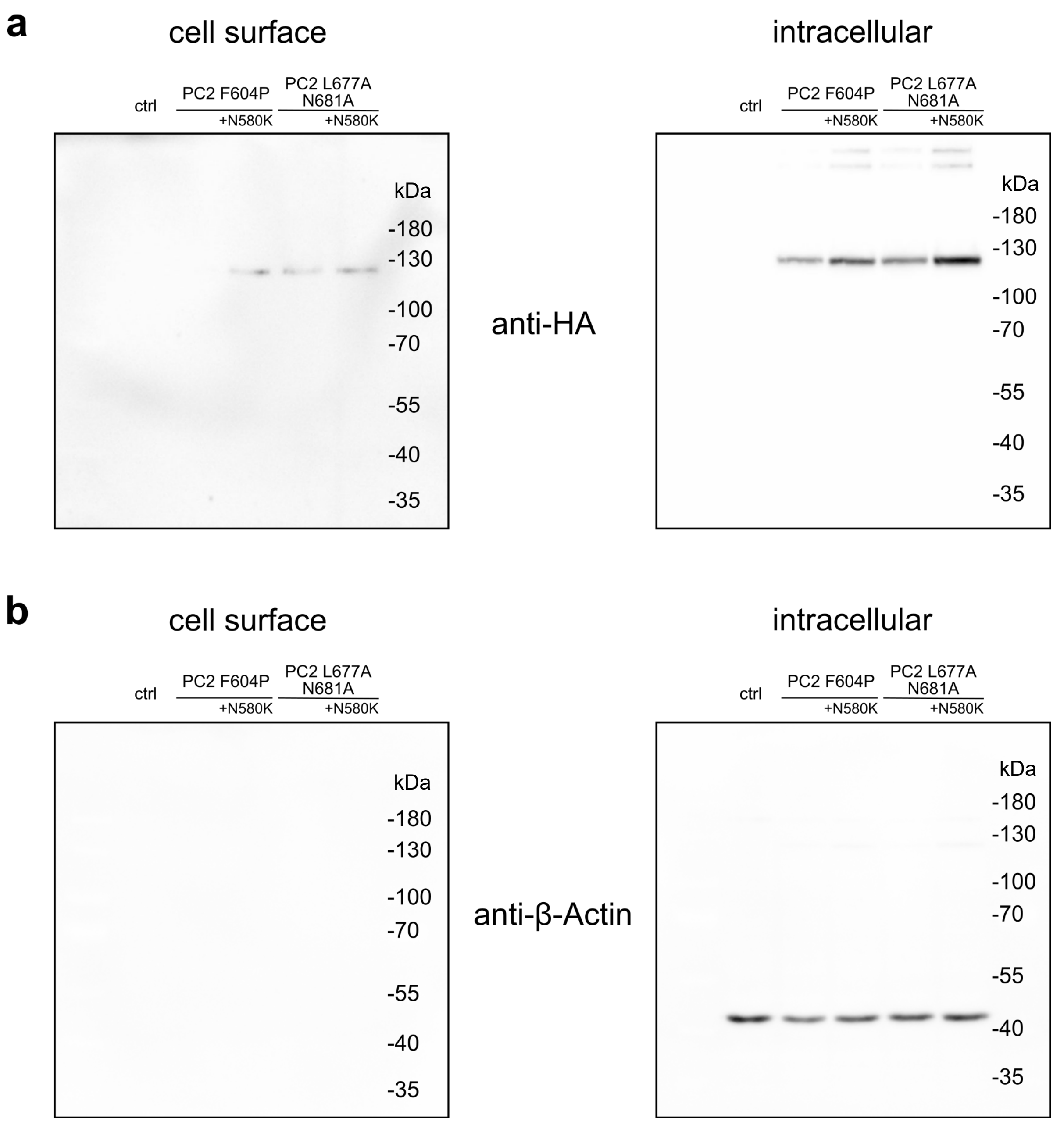

Fig. S7 Western blot analysis of PC2 constructs with the N580K mutation (oocyte batch 2) Cell surface (*left panel*) and intracellular (*right panel*) expression of PC2 mutants was detected using an antibody against the N-terminal HA-tag. **b**, To confirm separation of cell surface proteins from intracellular proteins, the membranes were stripped and re-probed using an anti-β-actin antibody.

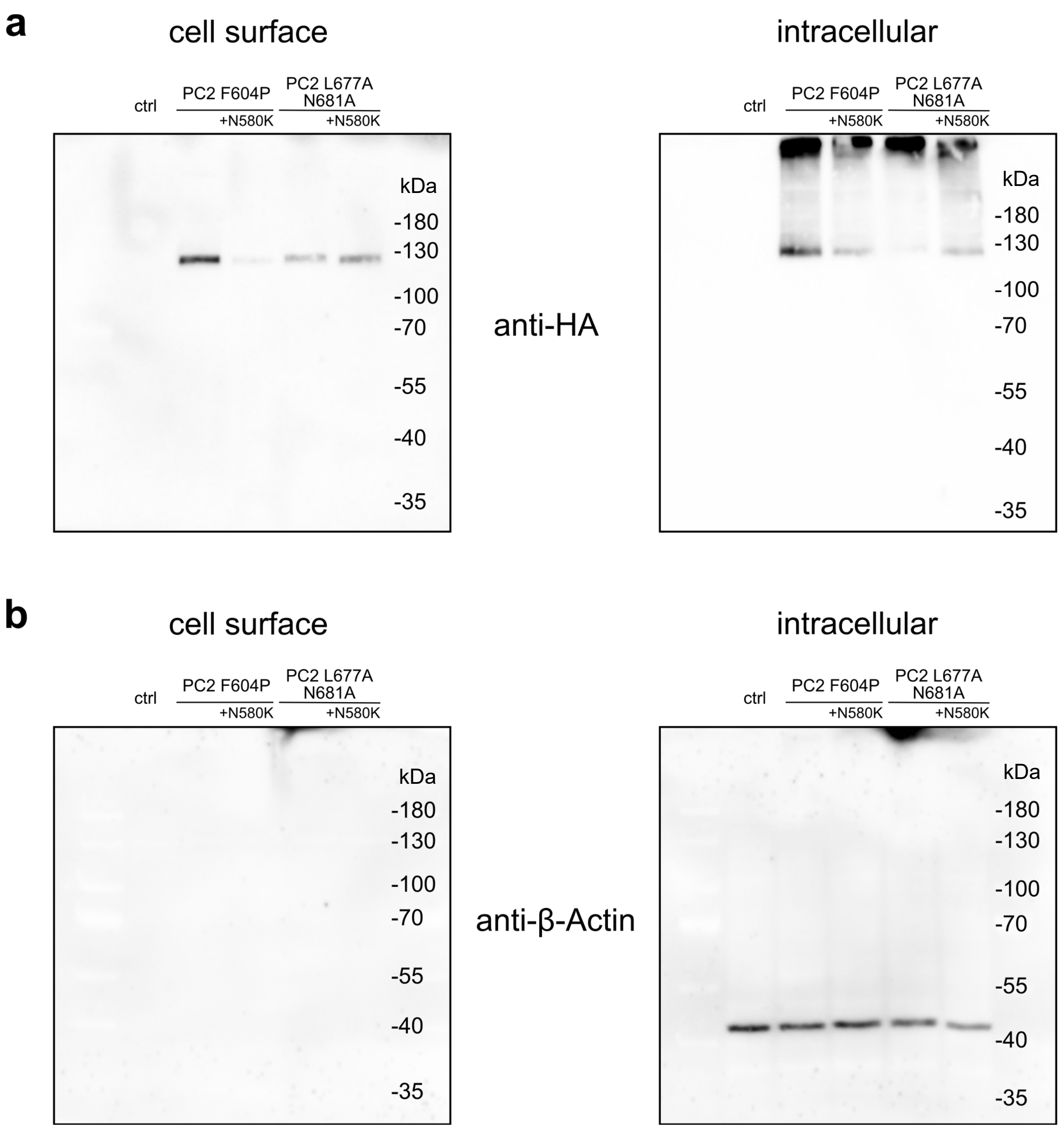

Fig. S8 Western blot analysis of PC2 constructs with the N580K mutation (oocyte batch 3) Cell surface (*left panel*) and intracellular (*right panel*) expression of PC2 mutants was detected using an antibody against the N-terminal HA-tag. **b**, To confirm separation of cell surface proteins from intracellular proteins, the membranes were stripped and re-probed using an anti-β-actin antibody.

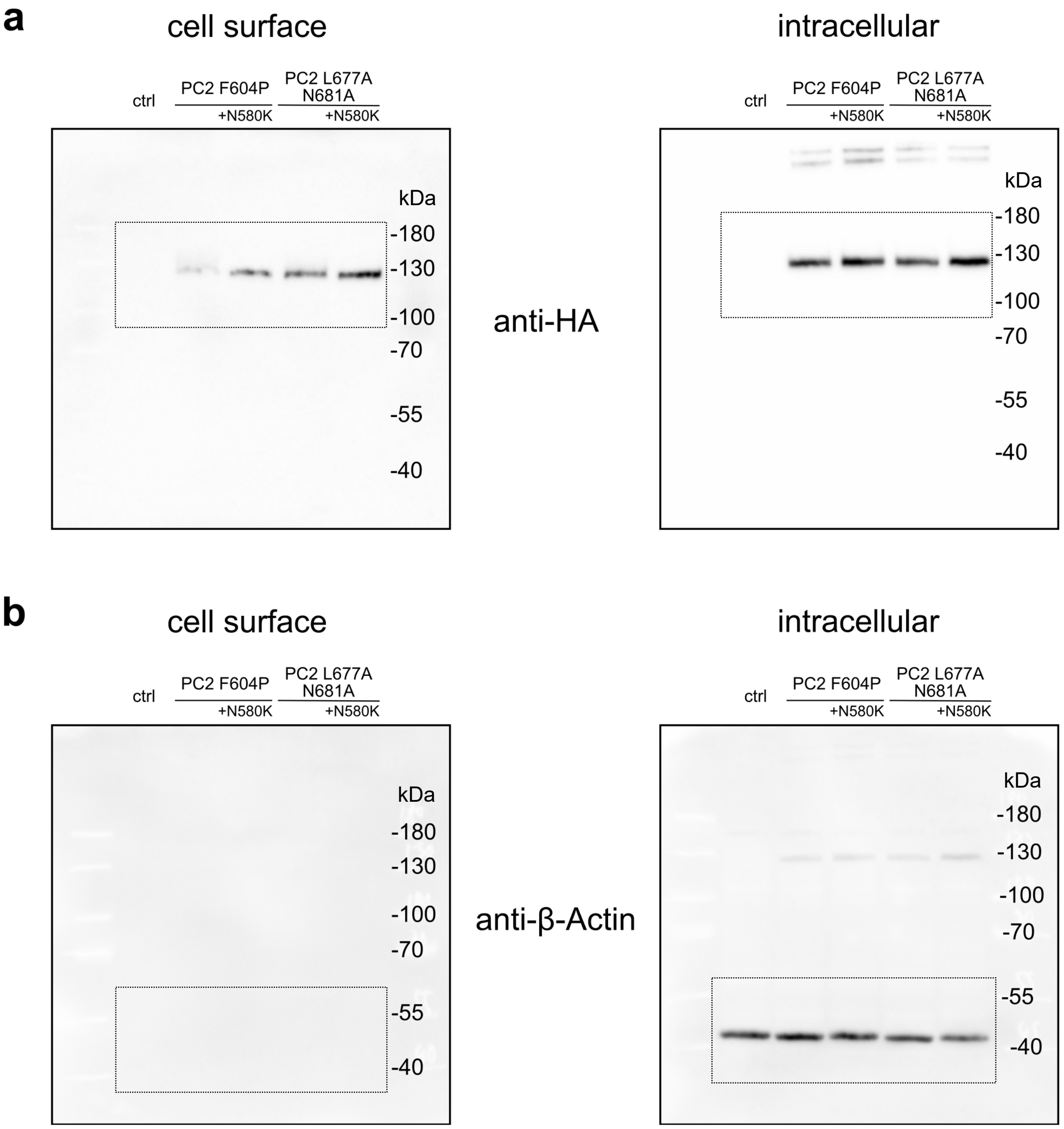

Fig. S9 Western blot analysis of PC2 constructs with the N580K mutation (oocyte batch 4) Original uncropped images of the same western blots shown in Figure 12**d**. Cell surface (*left panel*) and intracellular (*right panel*) expression of PC2 mutants was detected using an antibody against the N-terminal HA-tag. **b**, To confirm separation of cell surface proteins from intracellular proteins, the membranes were stripped and re-probed using an anti-β-actin antibody. Parts of blots shown in Fig. 12**d** are framed by dotted rectangles.

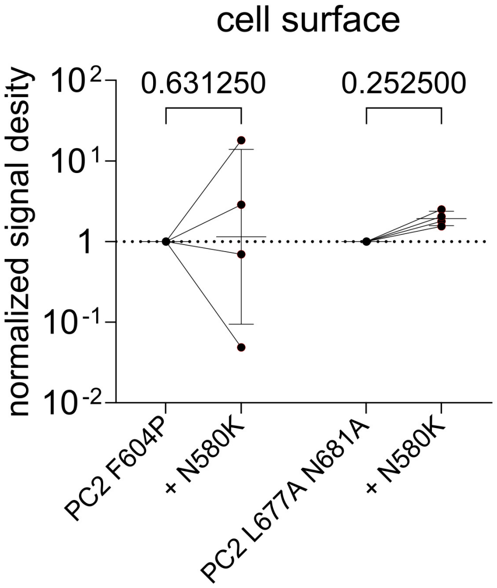

Fig. S10 The N530K mutation did not significantly affect PC2 expression at the cell surface. Densitometric analysis of the western blots shown in Figures S6a-S9a. In each blot, the signal density of the PC2 GOF construct with the N580K mutation was normalized to the corresponding signal density of the PC2 GOF construct without this mutation. *P-values* were calculated using Multiple Wilcoxon matched-pairs signed rank test.
